# The TCP transcription factor HvTB2 heterodimerizes with VRS5 and controls spike architecture in barley

**DOI:** 10.1007/s00497-022-00441-8

**Published:** 2022-03-07

**Authors:** Tatiana de Souza Moraes, Sam W. van Es, Inmaculada Hernández-Pinzón, Gwendolyn K. Kirschner, Froukje van der Wal, Sylvia Rodrigues da Silveira, Jacqueline Busscher-Lange, Gerco C. Angenent, Matthew Moscou, Richard G. H. Immink, G. Wilma van Esse

**Affiliations:** 1 Cluster of Plant Developmental Biology, Laboratory of Molecular Biology, Wageningen University and Research, 6708 PB Wageningen, The Netherlands; 2 Bioscience, Wageningen Plant Research, Wageningen University and Research, 6708 PB Wageningen, The Netherlands; 3 Laboratório de Biotecnologia Vegetal, Centro de Energia Nuclear na Agricultura, Universidade de São Paulo, Piracicaba, SP CEP 13416-000 Brazil; 4 The Sainsbury Laboratory, Norwich Research Park, Norwich, NR4 7UH UK; 5 Institute of Crop Functional Genomics, Rheinische Friedrich-Wilhelms-Universität, Bonn, Germany

**Keywords:** Barley, VRS5, Protein–protein interactions, TCP, HvTB2

## Abstract

**Key message:**

Understanding the molecular network, including protein-protein interactions, of VRS5 provide new routes towards the identification of other key regulators of plant architecture in barley.

**Abstract:**

The TCP transcriptional regulator TEOSINTE BRANCHED 1 (TB1) is a key regulator of plant architecture. In barley, an important cereal crop, HvTB1 (also referred to as VULGARE SIX-ROWED spike (VRS) 5), inhibits the outgrowth of side shoots, or tillers, and grains. Despite its key role in barley development, there is limited knowledge on the molecular network that is utilized by VRS5. In this work, we performed protein–protein interaction studies of VRS5. Our analysis shows that VRS5 potentially interacts with a diverse set of proteins, including other class II TCP’s, NF-Y TF, but also chromatin remodelers. Zooming in on the interaction capacity of VRS5 with other TCP TFs shows that VRS5 preferably interacts with other class II TCP TFs in the TB1 clade. Induced mutagenesis through CRISPR–Cas of one of the putative VRS5 interactors, HvTB2 (also referred to as COMPOSITUM 1 and BRANCHED AND INDETERMINATE SPIKELET 1), resulted in plants that have lost their characteristic unbranched spike architecture. More specifically, *hvtb2* mutants exhibited branches arising at the main spike, suggesting that *HvTB2* acts as inhibitor of branching. Our protein–protein interaction studies of VRS5 resulted in the identification of *HvTB2* as putative interactor of VRS5, another key regulator of spike architecture in barley. The study presented here provides a first step to underpin the protein–protein interactome of VRS5 and to identify other, yet unknown, key regulators of barley plant architecture.

**Supplementary Information:**

The online version contains supplementary material available at 10.1007/s00497-022-00441-8.

## Introduction

Plant architecture is a major determinant for yield and as such has been a target during domestication and breeding. In maize (*Zea mays*) the gene *TEOSINTE BRANCHED1 (TB1)* has been selected during domestication for its role in shaping plant architecture. TB1 inhibits the outgrowth of lateral branches and increased expression of *TB1* in maize resulted in a drastic reduction in number of branches and increased crop yield (Doebley et al. 1997; Hubbard et al. 2002). To date, *TB1* orthologs have been targeted for their effect on improved yield in several crops including, pea, potato, barley, rice and wheat (Takeda et al. 2003; Ramsay et al. 2011; Braun et al. 2012; Nicolas et al. 2015; Dixon et al. 2018). *TB1* is a member of the plant specific TCP transcription factor family. The family name refers to the founding members *TB1* in maize, *CYCLOIDEA (CYC)*, which is involved in controlling floral bilateral symmetry in snapdragon, and *PROLIFERATING CELL FACTORS (PCF1 and 2)* in rice (Luo et al. 1996; Kosugi and Ohashi 1997). PCFs bind to the promoter of *PROLIFERATING CELL NUCLEAR ANTIGEN (PCNA)* to control cell cycle in meristems, as well as DNA synthesis and repair (Kosugi and Ohashi 1997). This class of TF exhibits a highly conserved TCP domain, which contains a basic Helix–Loop–Helix (bHLH) structure involved in DNA binding and protein–protein interactions (Kosugi and Ohashi 1997; Cubas et al. 1999). The TCP transcription factor family can be divided into two major phylogenetic clades, class I and class II. TCPs play crucial roles in controlling plant architecture (Yuan et al. 2009; Lyu et al. 2020). In cucumber, for example, mutations in the TB1-clade TCP protein TEN, which contains a highly conserved amino acid sequence only found in Cucurbitaceae, resulted in plants that developed shoots instead of tendrils (Wang et al. 2015). The maize TB1-clade gene *BRANCHED ANGLE DEFECTIVE 1 (BAD1)* is required for normal tassel branch angle formation (Bai et al. 2012). The closely related gene in rice, known as *OsTB2* and as *RETARDED PALEA 1, REP1*) controls palea development and floral zygomorphy (Yuan et al. 2009). TB1, which acts as inhibitor of axillary meristem outgrowth (Corbesier et al. 2007; Aguilar-Martínez et al. 2007; Finlayson 2007; Kebrom et al. 2010, 2013), appears to be the most conserved member within the TCP TF family. In barley, *VRS5,* also referred to *as INTERMEDIUM-C (INT-C)*, is a key regulator of plant architecture and yield (Ramsay et al. 2011). *VRS5* is an orthologue of the maize domestication gene *TB1*. Barley grains (seeds) are formed on the inflorescence, which contains the grain producing florets that are arranged on a single main stem, the rachis (Koppolu and Schnurbusch 2019). The rachis develops specialized branches called spikelets, which eventually develop into seeds located on opposite sides of the rachis. Modifications to the overall spike architecture have been vital for cereal domestication and yield improvement (Boden and Østergaard 2019; Gauley and Boden 2019). In barley, the main spike (inflorescence) also underwent significant changes in architecture. For example, wild barley shatters the seeds from the main spike, a characteristic that was lost during domestication of barley (Pourkheirandish et al. 2015). To date, the barley spike occurs in two main architectural shapes: two-rowed or six-rowed. In two-rowed lines, only the central floret develops into a seed, in contrast to six-rowed cultivars in which all three florets develop into seeds. *VRS5* acts as inhibitor of lateral seed outgrowth. As such, allelic variation in *VRS5* has been selected in six-rowed barley cultivars for its role in shaping spike architecture (Ramsay et al. 2011). Detailed phenotypical analysis showed that *vrs5* mutants also exhibit an increased tiller number at early developmental stages (Ramsay et al. 2011; Liller et al. 2015; Zwirek et al. 2019). This suggests that, similar to its maize counterpart VRS5 inhibits the outgrowth of lateral branches.

Despite the key roles of VRS5 in barley development, there is hardly any knowledge on the molecular network in which VRS5 is active.. Here, we performed a comprehensive analysis of barley *TCP* genes and their chromosomal location. In total, we identified 21 barley *TCPs*: 11 class I and 10 class II. Given the key roles of TB1 in plant development, we focused on VRS5 and performed an unbiased Y2H screen to identify potential protein–protein interactors and to shed light on its molecular mode of action. This analysis was followed by a more detailed analysis of candidate interactors within the class II TCP TF family. We generated a CRISPR–Cas9-induced mutation in one of the genes encoding a putative VRS5 interactor, *HvTB2*. Our data show that barley plants that do not have a functional HvTB2 develop spikes that lose their characteristic determinate growth pattern. We also demonstrate that *HvTB2* is the gene underlying the COMPOSITUM 1 (COM1) locus, corroborating recent reports (Shang et al. 2020; Poursarebani et al. 2020). Taken together, our analysis shows that VRS5 has the capacity to heterodimerize with other transcriptional regulators, including closely related class II TCPs. Phenotypical analysis of one of the putative interactors shows that other class II TCPs, besides VRS5, are involved in controlling spike architecture in barley.

## Results

### Barley class II TCPs have a grass-specific sister clade of TB1

**Fig. 1:**
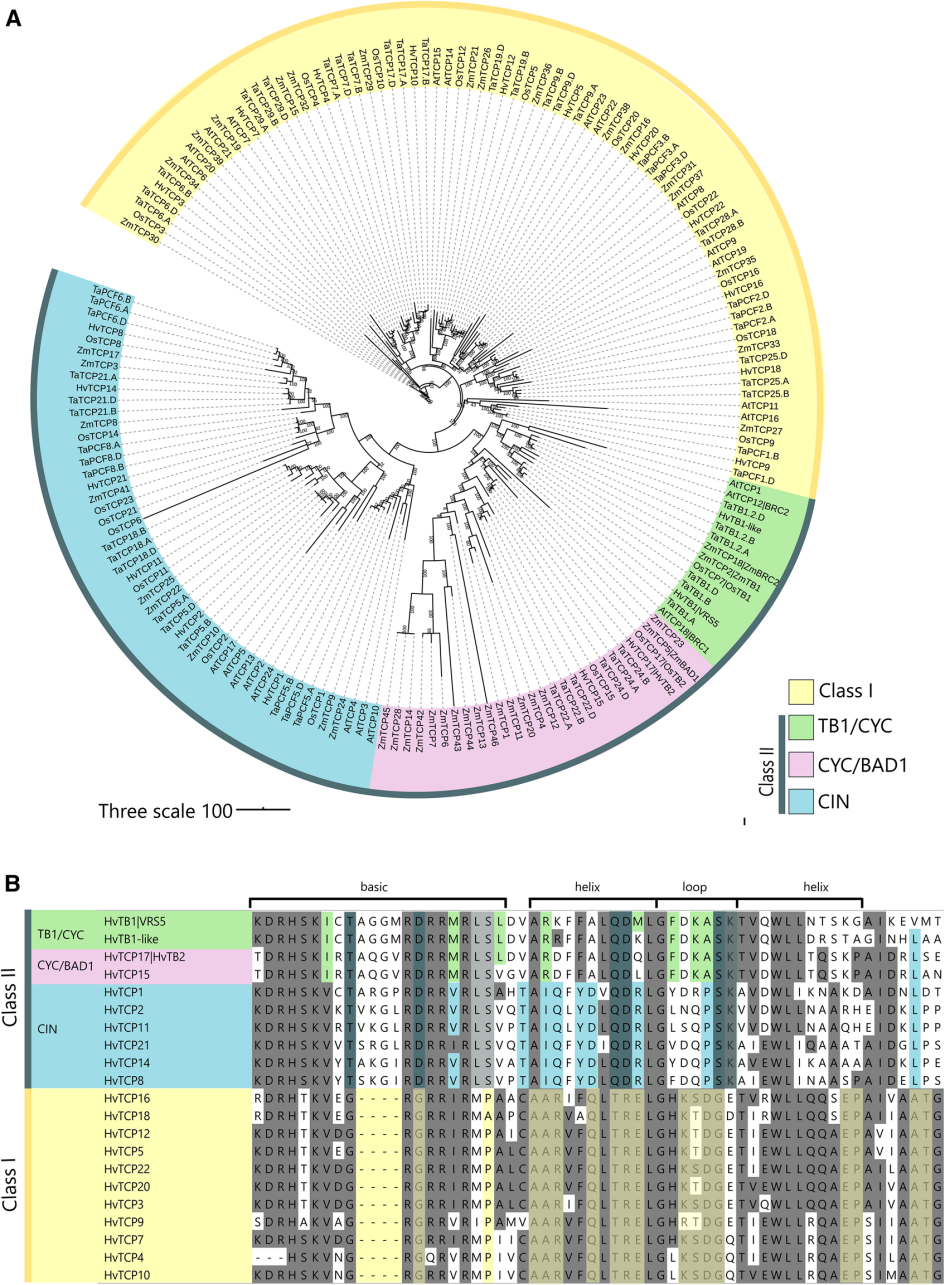
Phylogenetic relationships and sequence conservation of barley TCP transcription factors. **A** Maximum likelihood phylogenetic tree of TCP transcription factors from barley, wheat, rice, maize and Arabidopsis. **B** Amino acid sequence alignments of the TCP domain of barley TCPs. A gray background indicates a high similarity in the conserved TCP domain, independent of class I or II; green blue and yellow background indicates conserved amino acids corresponding to the TB1/CYC, CIN and class I clade, respectively. Purple indicates the two TCPs HvTB2 and HvTB15, which are most closely related to BAD1 according to the phylogenetic analysis

To elucidate the phylogenetic relations of the barley TCPs, a maximum likelihood (ML) phylogenetic tree was built including all known members of the barley, wheat, Arabidopsis, rice and maize TCP transcription factor families. With exception of wheat and barley, all sequences were extracted from the iTak (Zheng et al. 2016) and GRASSIUS database (Gray et al. 2009). Wheat TCP genes were extract from Zhao et al*.* (2018). For barley, the TCPs were extracted using a blastP search against the Morex V2 genome version, which was the most recent genome available at the time of the analysis (Monat et al. 2019). For completeness, gene ID numbers of barley TCPs in different genome versions within the Barlex database (Mascher et al. 2013, 2017, 2021; Colmsee et al. 2015; Monat et al. 2019) are included in Supplementary Table 1. The multiple sequence alignment was manually curated and non-aligning sequences were removed. For wheat, this included TaPCF7.A, TaPCF7.B, TaPCF7.D, which did not contain a TCP domain; and TaTCP19, which was partially truncated. In total 21 barley TCPs, 22 rice TCPs, 24 Arabidopsis TCPs, 62 wheat TCPs and 46 maize TCPs were included in the analysis (Supplementary Table 2). Similar to the situation in other plants, barley TCPs grouped into two main classes, class I (PCF) and class II (CIN/CYC/TB1) (Fig. 1), confirming the results of a recent analysis (Gao et al. 2021). Overall, TCPs were expressed in various tissue types and developmental stages (Supplementary Fig. 1) (Thiel et al. 2021). Barley TCPs have a close phylogenetic relationship to hexaploid wheat, which contains three copies of each TCP on the A, B and D genomes. Similar to wheat (Zhao et al. 2018), the TB1 locus is duplicated in barley, with a copy on chromosomes 4, and 5 (Fig. 1A, Supplementary Table 1). VRS5 and TaTB1, both located on chromosome 4, are known regulators of inflorescence architecture. However no function has been attributed to their paralogs, HvTB1-like [HORVU5Hr1G000490; (Walla et al. 2020)] and TaTB1.2, on chromosome 5.

Barley HvTB2 and HvTCP15 fall together with ZmBAD1 and OsTB2 into a sister clade of TB1 (Fig. 1A). To further elucidate the origin of this subclade, we performed a phylogenetic analysis comparing HvTB-like genes in 19 monocot and eudicot plant species. This analysis shows that both HvTB2 and HvTCP15 fall into a grass-specific sister clade of TB1 (Supplementary Fig. 2). Within this clade, HvTB2 is more similar to ZmBAD1 and to OsTB2, while HvTB15 is most similar to OsTCP15 and the sorghum *mutliseeded1* (msd1) TF, which is well known for regulating inflorescence architecture (Jiao et al. 2018). Taken together, similar to maize and rice, the barley and wheat TCP TF families have a grass-specific sister clade.

### VRS5 forms heterodimers with closely related class II TCP TF

**Fig. 2:**
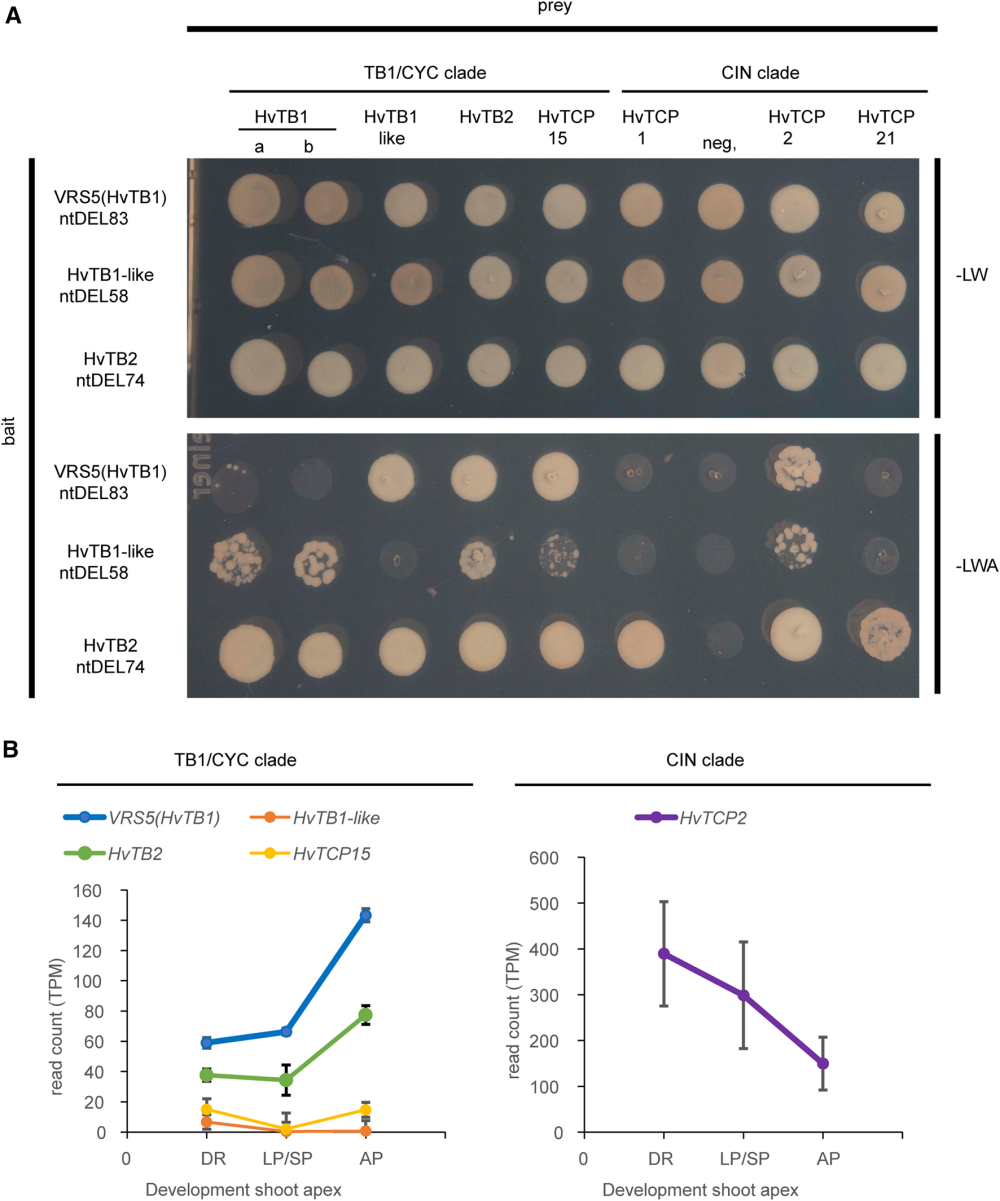
Protein–protein interaction and gene expression of barley class II TCPs. **A** Protein–protein interactions of barley TCP transcription factors (TF) in the TB1-clade and CIN-clade. Interactions were scored on the medium lacking leucine (L), tryptophan (W) and adenine (A), medium lacking L and W was used as positive control for the mating. As negative control (neg.) a barley gene annotated as TF with unknown function was used as prey. For the bait vector, N-terminal deletion constructs of VRS5, HvTB1-like and HvTB2 were used. **B** Expression of TCPs that interact with VRS5 based on transcripts per million (TPM). Expression data obtained from GSE102191 (van Esse et al. 2017) and GSE149110 (Walla et al. 2020). *DR* double ridge stage, *LP/SP* the lemma and stamen primordia stage, *AP* the awn primordium stage

TCP transcription factors can form homo- and heterodimers, which affect their DNA binding capacity and specificity. To evaluate the protein–protein interaction capacity of barley VRS5 we performed unbiased and targeted yeast two-hybrid (Y2H)-based screenings using this TCP protein as bait. Because of autoactivation of yeast reporter genes, the N-terminal part of the HvTB1 protein was removed VRS5^NtDEL83^. Subsequently, we generated a Y2H cDNA expression library of the early and late developmental stages of the barley shoot apical meristem (SAM), respectively (Supplementary Fig. 3). These stages were selected as VRS5 is highly expressed in the developing SAM (Fig. 2B). Screening of VRS5^NtDEL83^ against the barley cDNA libraries resulted in the identification of 114 positive colonies, from which 16 encoded unique proteins in frame with the GAL4 AD-domain (Supplementary Table 3). Amongst these are SWItch/Sucrose Non-Fermentable (SWI/SNF) complex subunit (HORVU5Hr1G037060.1), NUCLEAR TRANSCRIPTION FACTOR Y (NF-Y; HORVU6Hr1G032200.3) and HvTCP2 (HORVU3Hr1G073830.1), which are all highly expressed in the developing shoot apical meristem (Supplementary Table 3). It is well known that TCP proteins interact amongst each other with a preference for interaction with other members within the same clade (Danisman et al. 2013). However, interactions can be easily missed in a library screening. Therefore, we decided to evaluate the interaction between VRS5^NtDEL83^ in the BD vector against a Arabidopsis TF Y2H library (Pruneda-Paz et al. 2014) in a heterologous Y2H screen. In this screen, we identified AtTCP1 (AT1G67260) and AtBRC1 (AT3G18550), both class II TCPs, as interactors. No interaction was observed with any of the class I TCP proteins, as expected. Within the heterologous screen, we also observed an interaction with AtNF-Y proteins, which confirms the interaction found with barley NF-Y factors in the barley cDNA library screen. Additionally, VRS5 was capable of interacting with Arabidopsis HOMEODOMAIN-containing proteins, and MYB-like transcriptional regulators (Supplementary Table 3).

To further evaluate protein interactions of VRS5, we investigated its capacity to form complexes with other class II TCPs in barley. For this, we selected as preys: HvTCP1, HvTCP21 and HvTCP2 which belong to the CIN clade; and the four class II TCPs within the TB1 and CYC/BAD1 clade (Fig. 1). In this targeted analysis, two VRS5 protein variants were included, encoded by two natural alleles, the a and b alleles, which correspond to the six-rowed and two-rowed cultivars, respectively (Ramsay et al. 2011). Because of autoactivation by the selected class II TCP proteins, no complete pair-wise matrix-based screen could be performed. For this reason, we generated N-terminal deletion variants for VRS5, HvTB1-like and HvTB2 and used these truncated proteins as baits. VRS5 and HvTB1-like showed a weak homo- and heterodimerization capacity (Fig. 2A, Supplementary Fig. 4). No difference in this homo- and hetero dimerization capacity was observed between the a- and b allele variants of VRS5. Both VRS5 and TB1-like proteins showed a consistent interaction with HvTB2, HvTCP15 and HvTCP2 (Fig. 2A). Vice versa, HvTB2 interacted with both VRS5 and HvTB1-like. No interaction was observed between VRS5 or HvTB1-like and the CIN-clade proteins, HvTCP1 and HvTCP21. Altogether these experiments revealed that the barley TB1-like TCPs preferentially interact with closely related members within the class II clade of TCP proteins.

For biological relevance, genes encoding interacting proteins should be co-expressed and therefore, we compared the expression patterns and levels of *VRS5* and of the genes encoding the interacting TCP TF in the developing shoot apex, extracted from available RNA-Seq data of cv Bowman apical meristems (van Esse et al. 2017; Walla et al. 2020; Thiel et al. 2021). Both *VRS5* and *HvTB2* have a low expression at the double ridge stage, their expression increases in the lemma and stamen primordia stages (LP/SP) and is highest in the awn primordia stage (AP) (Fig. 2B, Supplementary Table 4). In comparison, *HvTB1-like* and *HvTCP15* are lowly expressed within the developing shoot apex at all three investigated developmental stages. *HvTCP2* is highly expressed in the shoot apex, but follows an opposite trend in time when compared to *VRS5* and *HvTB2*. Recently, it was shown that *VRS5* is higher expressed in the lateral spikelet when compared to the central spikelets (Thiel et al. 2021). A more closer look at the *HvTB2* expression suggests that this gene is highly expressed in both the central and the lateral spikelets within the developing shoot apex (Supplementary Fig. 1). Taken together, HvTB2 follows a similar expression pattern as its putative interaction partner VRS5.

### Barley HvTB2 controls spike branching

**Fig. 3:**
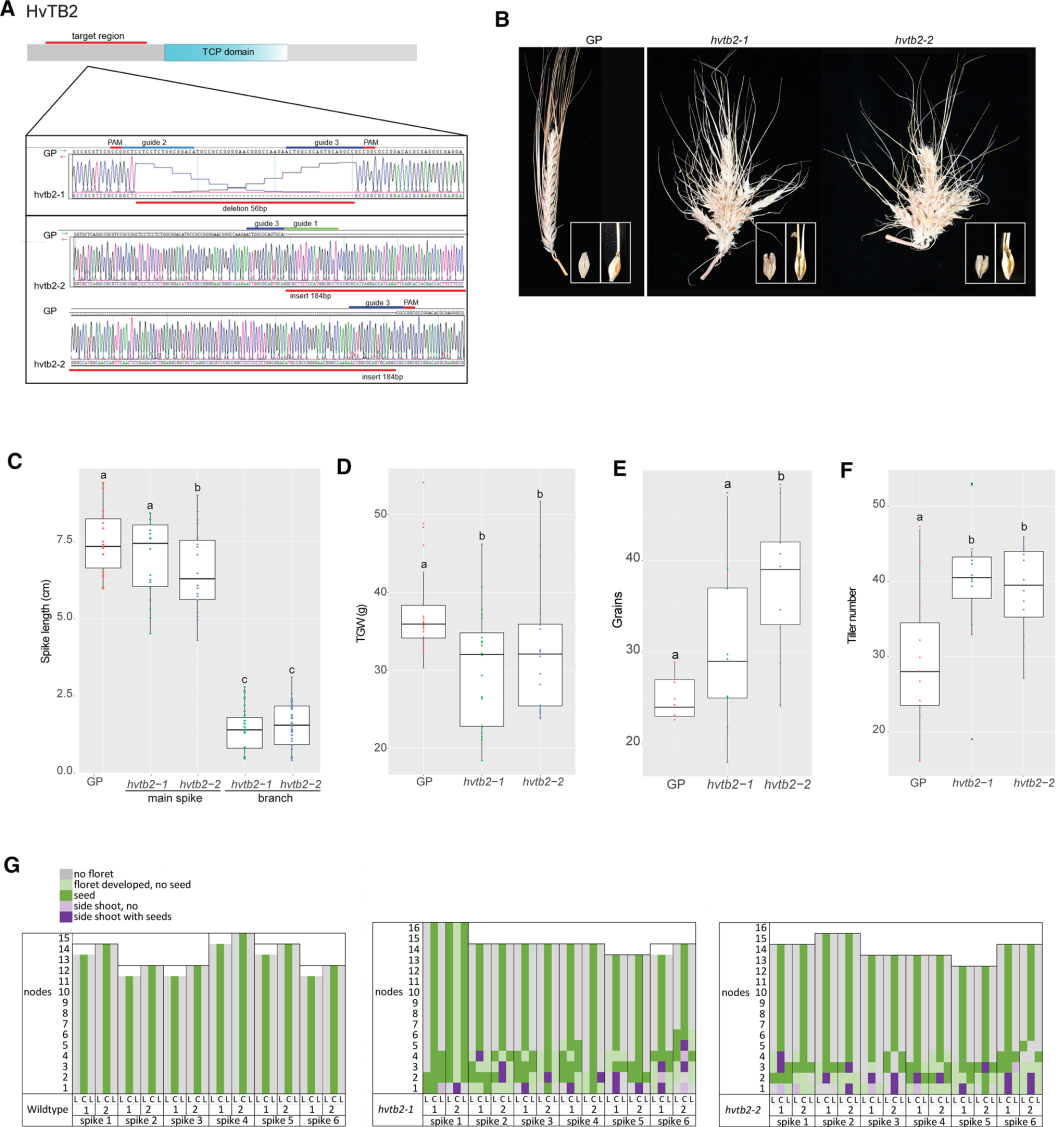
Macroscopic phenotype of HvTB2 mutants.** A** CRISPR–Cas9 target site and *hvtb2* mutants generated. Trace files show the sequence of cv. Golden Promise (GP) in comparison the 57 bp deletion mutant, *hvtb2-1* and the 184 bp insertion mutant *hvtb2-2*. **B** Spike phenotype of the wild-type GP in comparison to the generated *hvtb2-1* and *hvtb2-2* mutants. Right corner inset shows an enlarged image of the seeds, with clear split of the awn and fused seeds which is observed in both mutants. **C**–**F** Spike length, thousand grain weight (TGW), number of grains per spike and tiller number measurements of GP, *hvtb2-1* and *hvtb2-2*. Per genotype: spike length *n* = 18 spikes; grains per spike *n* = 9 spikes; for tiller number *n* = 12 plants. TGW is based on extrapolation of the weight of 15 seeds, *n* = 20 pools. Different letters indicate experimental groups that were significantly based on a one-way ANOVA (*p* ≤ 0.05), same letters indicate not significant under this criterion. Spike length in GP was compared to *hvtb2-1* and *hvtb2-2*, for branch length, the comparison was made to the respective mutant spikes to *hvtb2-1* and *hvtb2-2* as normal wild-type GP does not have branches. **G** Quantification of the *hvtb2* spike phenotype. Seeds per rachis internode on each side of the spike are indicated in green. GP did not contain any lateral spikelets (gray) nor lateral branches while in *hvtb2* most of the basal lateral spikelets are developed into seeds (pink). Purple blocks indicate branches occurring at the rachis node. C indicates central spikelet, L indicate lateral spikelets

HvTB2 is a putative interactor of VRS5, and follows a similar expression pattern when compared to VRS5 (Fig. 2). Moreover, our phylogenetic analysis showed that HvTB2 is closely related to maize ZmBAD1 and OsTB2, with a similar domain architecture when compared to ZmBAD1 (Fig. 1). These observations prompted us to study the function of HvTB2 in more depth and led to the hypothesis that HvTB2 influences inflorescence architecture in barley, at least partially in concert with VRS5. To test this hypothesis, we generated targeted mutations in *HvTB2* using CRISPR–Cas9 gene editing in barley cv. Golden Promise (GP). Aiming at larger deletions and a specific null mutant for this TCP gene, three guides were used, all targeting the N-terminal part of *HvTB2* before the conserved TCP domain (Fig. 3A, Supplementary Fig. 5). In total, 38 Cas9-positive plants were generated, from which one showed a putative biallelic event. Screening of the T2 transformants of this line resulted in two novel *HvTB2* alleles, *hvtb2-1* and *hvtb2-2*, containing a 56 bp deletion and a 184 bp insertion, respectively (Fig. 3A). In both *hvtb2-1* and *hvtb2-2,* the mutational event caused a frame shift before the TCP domain, thereby generating full null mutants of *HvTB2*. Both mutants exhibited spikes that lost the characteristic determinant growth pattern, with branches forming on the main rachis (Fig. 3B). The seed-bearing branches were significantly shorter when compared to the main spike (Fig. 3C, Supplementary Table 5). The outgrowth of branches from the main rachis occurred mainly on the basal part of the spike (Fig. 3G). In addition, we also observed, that some of the basal seeds in *hvtb2* showed two awns and/or fused seeds, a phenotype that does not occur in the wild-type GP (Fig. 3B). This fused seed phenotype was observed in about 10% of the seeds (Supplementary Fig. 6C). Due to the reduced spike length, the total number of grains was only moderately increased in the *hvtb2* lines, despite the presence of lateral branches (Fig. 3E). The thousand grain weight (TGW) and grain width were significantly reduced in the *hvtb2*-lines (Fig. 3D; Supplementary Fig. 6D, E). Overall, the grain size in the lateral branches was reduced when compared to the main spike (Supplementary Fig. 6D, E). Interestingly, *hvtb2* mutants displayed a minor, but significant, increase in tiller number when compared to the wild type GP (Fig. 3F). Taken together, our data suggest that *HvTB2* influences multiple yield-related traits throughout barley development, similar to VRS5. The macroscopic phenotype resembles previously described phenotype for *intermedium-h* (*int-h*) and *compositum 1* (*com1*) (Druka et al. 2011; Franckowiak et al. 2020). Targeted PCR amplification showed no amplicon in the coding sequence or promoter region of *int-h42*, *int-h.43* and *int-h.44, int-h.83*, *com1.a* and *com1.b* (Supplementary Fig. 7). Two of the lines tested, *int-h.83* and *com1.c* contained a nonsynonymous mutation that resulted in an amino acid change within the conserved TCP domain (Supplementary Fig. 8). Therefore, *HvTB2* is a good candidate gene for the *int-h* and *com1.a* loci, corroborating recent reports (Poursarebani et al. 2020). Taken together, we identified *HvTB2* as gene controlling spike architecture.

### hvtb2 acts as a boundary gene

**Fig. 4:**
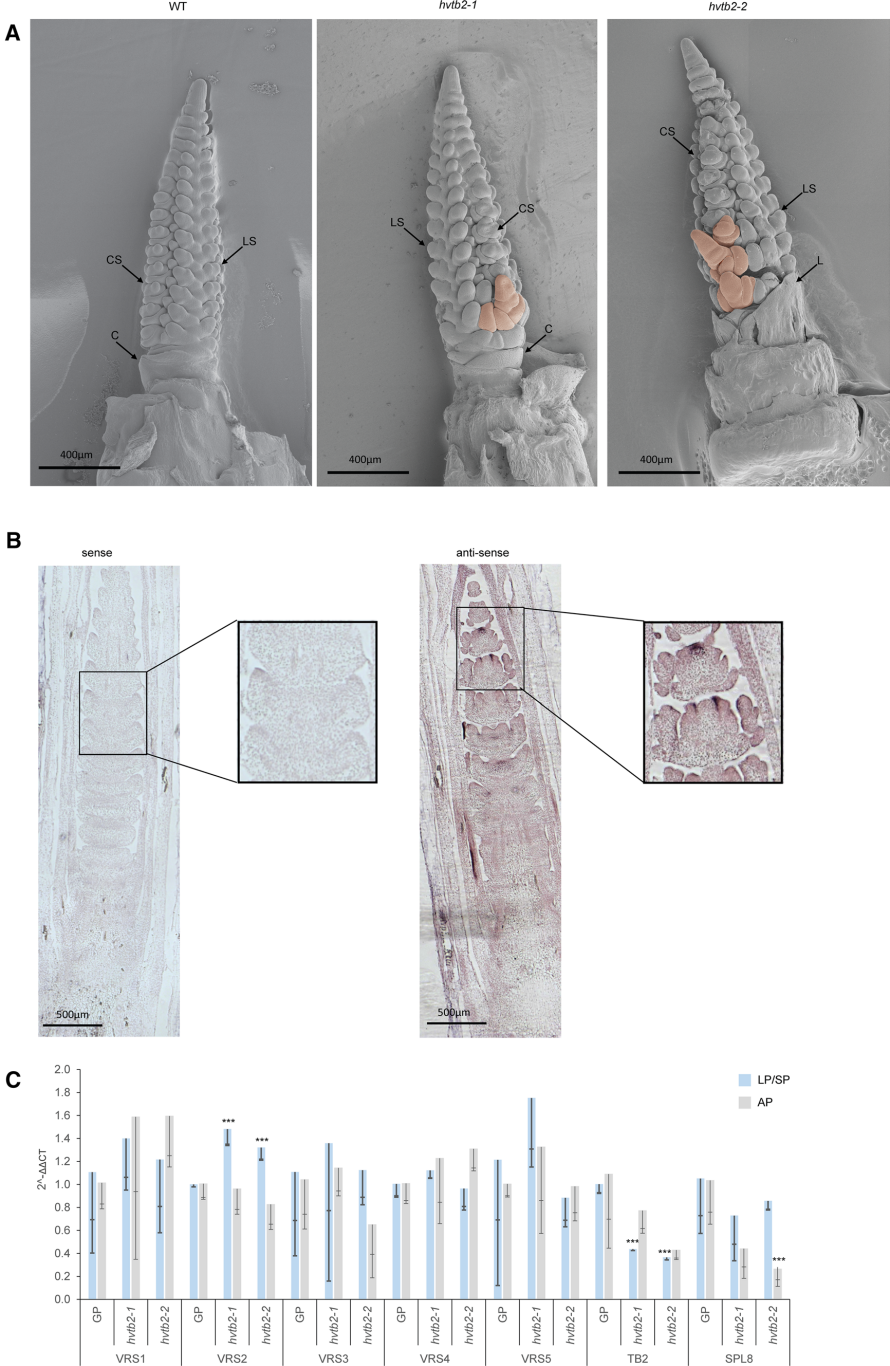
Meristem phenotype of *hvtb2* mutants. **A** Scanning electron microscope images taken at the lemma and stamen primordia stages (LP/SP). Pink color indicates the outgrowing branch structure in the developing meristem. *CS *central spikelet meristem, *LS* lateral spikelet meristem, *C* collar, *L* leaf. **B** RNA in situ hybridization of *HvTB2* in the background of cv Bowman. Squares in panel are enhanced images in the defined region. **C** RT-PCR analysis of the VRS genes, TB2 and SPL8 in *hvtb2-1* and *hvtb2-2* compared to the wild type cv Golden Promise (GP) at the LP/SP and AP. Statistical differences between the ΔΔ*C*_T_ values was calculated using a *t* test using a *p* value of 0.05 as the threshold. Asterisks indicate significant differences when compared to GP. For all data-points *n* ≥ 3 biological replicates

To determine the origin of the lateral branches that appear on the main rachis we compared the morphology of wildtype GP and *hvtb2* mutants at LP/SP using scanning electron microscopy. In GP the triple spikelet meristem is formed and outgrowth of lateral branches is supressed. In contrast, the central spikelet at the base of the meristem of *hvtb2* mutants is enlarged, resembling a branch meristem instead of a triple spikelet meristem (Fig. 4A). This altered development mainly occurs at the basal part of the spike. In line with this the branches in the mature spike are only observed in the first five rachis nodes (Fig. 3G). Overall, no major differences were observed in leaf number or the overall developmental speed of the apex (Supplementary Fig. 9), suggesting that HvTB2 mainly acts on inhibition of the spike branching. The lateral spike branch showed an indeterminate growth pattern, and continued to grow and differentiate after producing the floret meristems. The branch meristem-like structures are still vegetative at the stamen primordium stage (Fig. 4A), and start to initiate spikelet primordia when the inflorescence transitions to the awn primordium stage (Supplementary Fig. 9). No major phenotypes were observed at the double ridge stage (Supplementary Fig. 9A). In line with this, expression of *HvTB2* is low in this tissue and not yet localized to the spikelet primordia (Fig. 2B, Supplementary Fig. 10). RNA *in situ* hybridization shows that, at the awn primordium stage, *HvTB2* mRNA is mainly expressed at spikelet meristem boundaries (Fig. 4B, Supplementary Fig. 10). We did not observe an expression in the leaf axils. This suggest that HvTB2 may act within the triple floret meristem as boundary gene.

Next, we evaluated to what extent *HvTB2* influences the expression of other, known regulators of row-type architecture. To this end, we performed RT-PCR analysis in immature shoot apices of *tb2-1* and *tb2-2* mutants compared to wild type GP lines. Two developmental stages were selected, the lemma and stamen primordia stage (LP/SP) and the awn primordium stage (AP), where at the LP/SP a significant downregulation of *HvTB2* was observed. With exception of *VRS2*, which was significantly upregulated at the LS/SP stage, none of the other row-type genes was changed in expression in neither *tb2-1* nor *tb2-2* (Fig. 4C). We also included *SQUAMOSA PROMOTER-BINDING-LIKE8* (*SPL8;* HORVU0Hr1G039150) (Tripathi et al. 2018). In maize the SPL8-like gene *LIGULELESS 1* (*LG1*), acts downstream of ZmRAMOSA2 (RA2) and ZmBAD1 (Bai et al. 2012). Interestingly, SPL8 is significantly downregulated in the *hvtb2-2* mutant at the AP stage (Fig. 4C), suggesting that like in maize SPL8-like genes acts downstream of *hvtb2*. Taken together, our detailed phenotypical analysis indicates that *HvTB2* controls spike determinacy and acts as a boundary gene.

### Barley *HvTB2* is highly conserved

**Fig. 5:**
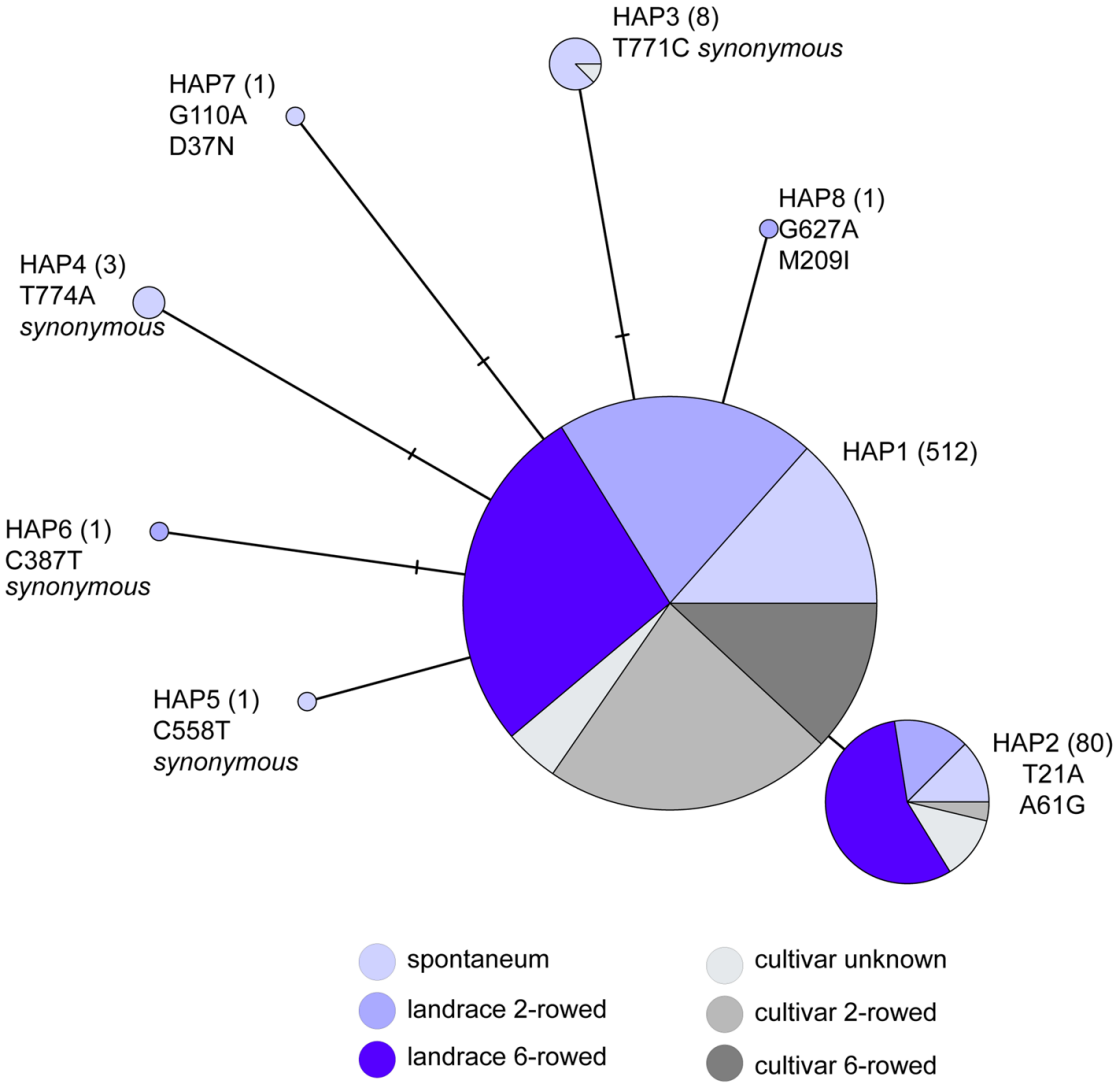
Haplotype analysis of *HvTB2*. Haplotype analysis is done on SNPs present in 607 individual plant lines ranging from *H. spontaneum*, landraces and cultivars with two-rowed or six-rowed spike architecture, using publicly available data sets (Russell et al. 2016; Bustos‐Korts et al. 2019). Number of plants per haplotype is indicated between brackets, SNPs identified and changes that occur at the amino acid level are stated below the haplotype

*TB1* is a well-known gene targeted during domestication of several crops including maize, wheat, rice and barley. To evaluate if *HvTB2* is also subjected to selection we performed a haplotype analysis based on available single-nucleotide polymorphism (SNP) (Russell et al. 2016; Bustos‐Korts et al. 2019). To assess both natural variation and possible selection through breeding, sequences from cultivars and landraces were included. For comparison, *VRS5* was also included in the analysis. Our analysis indicates that there are 4 major *VRS5* haplotypes. Two major haplotypes are primarily found in six-rowed and two-rowed cultivars, respectively (Supplementary Fig. 11), corroborating previous reports (Ramsay et al. 2011). Haplotype analysis on *HvTB2* shows two major haplotypes (HAP1 and HAP2), and six minor haplotypes. From these, four minor haplotypes did not cause a change in the amino acid sequence when compared to HAP1 (Fig. 5). For the other remaining haplotypes, no changes were observed in the conserved TCP domain. Based in the PROVEAN score for conservation analysis no functional changes are expected between the haplotypes (Supplementary Fig. 8B). None of the haplotypes identified were specific for either two-rowed or six-rowed cultivars nor for wild barley, landraces or cultivars (Fig. 5). Taken together, our results indicate that there is very little variation within the HvTB2 gene.

## Discussion

TCP transcription factors are essential for growth and development of plants and involved in a plethora of processes. They are a widespread family of transcriptional regulators occurring in multicellular algae, monocots and dicots (Navaud et al. 2007; Danisman 2016). In this study we performed a detailed phylogenetic analysis of barley TCP transcription factors and evaluated the protein–protein interactions of VRS5. One of the identified interactors and closely related protein, HvTB2 showed a similar expression pattern. Targeted mutagenesis showed that HvTB2 is essential for maintaining barley spike architecture. Taken together, this work increases our understanding of the role of TCP transcription factors in shaping barley plant architecture.

TCP transcription factors can form homo- and heterodimers, which affect their DNA binding capacity and specificity. They interact with a plethora of other proteins, including components of the circadian clock and various other transcriptional regulators (Danisman 2016; Bemer et al. 2017). Within the unbiased screen of VRS5 against the Y2H barley cDNA libraries we identified SWItch/Sucrose Non-Fermentable (SWI/SNF) complex subunits. At the protein level, the activity of CIN-like TCPs is known to be regulated by several chromatin remodeling complexes including SWI/SNF (Efroni et al. 2013; Sarvepalli and Nath 2018). The interaction of VRS5 with SWI/SNF might therefore point towards a conserved mechanism, where the activity of TB1 is modulated by chromatin remodeling factors at the protein level, similar to the CIN-like TCPs. We also identified other transcriptional regulators such as TCPs and NF-Y amongst the interactors of VRS5 in both the unbiased screen and the heterologous screen against the Arabidopsis TF collection. Large scale Y2H interaction studies in Arabidopsis showed an interaction between AtBRC1 with NFY9 (Trigg et al. 2017). NF-Y proteins are a large family of transcriptional regulators known to act in several plant developmental processes and abiotic stress responses (Petroni et al. 2013). It, therefore, remains to be evaluated how specific the interaction between VRS5 and members of the SWI/SNF chromatin remodeling and NF-Y TF family are. Nevertheless, our analysis shows a glimpse into the putative protein–protein interactome of VRS5. Together, this forms an ideal basis for possible future follow-up studies such as *in planta* confirmation of the interactions for targets of interest using co-immunoprecipitation (coIP) or Bimolecular fluorescence complementation (BiFC) combined with localization studies using *in situ* hybridization and genetic interactions.

Further, more detailed analysis using barley class II TCPs shows that within the class II TCPs VRS5 preferably heterodimerizes with the CYC/TB1 clade rather than the CIN clade. One of these key putative VRS5 interactors identified is HvTB2 which, similar to VRS5, inhibits tiller outgrowth. However, some difference in functionality also occurs. While VRS5 inhibits the outgrowth of lateral florets in the main spike through regulation of VRS1, HvTB2 does not show an obvious row-type phenotype. Instead, HvTB2 suppresses the outgrowth of branches from the main spike. This points towards a mechanism in which VRS5 and HvTB2 are only partially redundant. Taken together, VRS5 heterodimerizes with other transcriptional regulators. To what extent the heterodimerization of VRS5 influences DNA binding and subsequent transcriptional regulation of the target genes remains to be elucidated. Taken together, our analysis opens up the opportunity for expanding the VRS5 interactome and the subsequent identification of other key regulators of plant architecture such as HvTB2.

Genome duplication and diversification has played a major role in the evolution of the TCP transcription factor family. For example, mosses and ferns contain five to six members (Navaud et al. 2007), whereas the dicot model system Arabidopsis has 24 (Martín-Trillo and Cubas 2010). The gene duplication events are not always uniform, maize for example shows duplicates mainly in the CYC/TB1 clade. We identified 21 TCP transcription factors in barley and 62 in wheat. Wheat contains mostly three orthologues when compared to barley, representing the hexaploidy of the genome. Like in maize and rice, barley and wheat have additional grass-specific duplicates in the TB1/CYC clade. Within this clade both barley and wheat contain close homologues, such as maize *ZmBAD1* and rice *OsTB2* genes, *HvTB2* and *TaTCP24*, respectively. Although these genes are phylogenetically closely related, vast differences in functionality are observed. *ZmBAD1* (also referred to as *WAB1*) is expressed in the pulvinus where it regulates branch angle in the tassel(Bai et al. 2012; Lewis et al. 2014). *OsTB2* (also referred to as *REP1*) is involved in palea development and floral zygomorphy in rice. It is expressed in the palea primordium during early flower development and in later stages in the stamens and vascular bundles of the lemma and palea (Yuan et al. 2009; Lyu et al. 2020). Recent studies have shown that *OsTB2* is also expressed in the basal tiller node where it induces the outgrowth of tillers (Lyu et al. 2020). *OsTB1* and *OsTB2* act antagonistically on tiller development. *HvTB2* is mostly expressed in the developing inflorescence, where it follows a similar expression pattern when compared to *VRS5*. Targeted mutagenesis of *HvTB2* resulted in spikes that have lost their characteristic determinant growth pattern and exhibited lateral branches arising from the main spike. This suggests that *HvTB2*, in contrast to its rice homologue, acts as branching inhibitor rather than as inducer. In line with this, our phenotypic analysis showed that *hvtb2* mutants exhibited a minor, but significant, increased tiller number when compared to the wild type cv Golden Promise, potentially revealing a more general role as branching inhibitor. In this respect, HvTB2 does not appear to act antagonistically to HvTB1 on tiller development.

Haplotype analysis indicates that *HvTB2* is highly conserved in barley. Considering the phenotype of *hvtb2* it is highly tempting to speculate that *HvTB2* was under selection to maintain spike architecture. The function of *TaTCP24*, which is phylogenetically closely related to HvTB2 and also expressed in developing spikes (Zhao et al. 2018), remains to be elucidated. Taken together, although *BAD1, OsTB2* and *HvTB2* are phylogenetically closely related, they seem to exhibit functional diversity.

RNA in situ hybridization shows that *HvTB2* mRNA is localized at spikelet meristem boundaries. In addition, we observed presence of fused seeds in the generated CRISPR- *hvtb2* knockouts. Together, this suggests that HvTB2 plays a role in the specification of the spikelet meristem boundaries. Recently, two independent manuscripts were published while this work was under preparation (Shang et al. 2020; Poursarebani et al. 2020). In the first one, published by Shang et al. (2020), the *BDI1* locus was mapped, and the underlying gene corresponded to *HvTB2*. In that work, a significant upregulation of both *SPL8-like* and *HvTB2* was observed in the *vrs4* mutants, while in *hvtb2(bdi1) SPL8* was significantly downregulated at the awn primordium stage. Our RT-PCR analysis also shows that *HvSPL8-like* was significantly downregulated in the *hvtb2* mutants. This suggest that *HvTB2* acts upstream of *SPL8-like*, similar to maize *ZmBAD1(WAB1)* pointing to a conserved mechanism at the molecular level. In a second manuscript, published by Poursarebani et al (2020), it was shown that *HvTB2* is the causal gene underlying the *com1* and *int-h* locus, which is corroborated by our analysis. Previously, it was demonstrated that *VRS5* acts downstream of VRS4(HvRA2), a key regulator of row type which promotes spikelet and floret determinacy (Koppolu et al. 2013; van Esse et al. 2017; Zwirek et al. 2019). In maize, RA2 acts upstream of *ZmBAD1*, which is phylogenetically closely related to *HvTB2*. Like *HvTB2, VRS4* transcript is located in the boundary region (Koppolu et al. 2013). Poursarebani et al (2020), placed *HvTB2* downstream of *VRS4* and showed a down regulation of *HvTB2* in *vrs4.k* at the double ridge and AP/LP stage (Poursarebani et al. 2020). This suggests that *VRS4* acts upstream of both *VRS5* and *HvTB2*, at least in regulating inflorescence architecture. Functional *VRS4* prevents the outgrowth of lateral florets through activating *VRS5* and *VRS1,* the latter is a well-known conserved inhibitor of lateral floret development (Komatsuda et al. 2007). As such, *vrs1*, *vrs4* and *vrs5* single mutants display a six-rowed (*vrs1, vrs4*), or intermediate (*vrs5*), phenotype where lateral florets are developed (Komatsuda et al. 2007; Ramsay et al. 2011; Koppolu et al. 2013; Liller et al. 2015; Zwirek et al. 2019). Both *VRS4* and *VRS5* act on lateral floret development through modulating *VRS1* expression (Liller et al. 2015; Zwirek et al. 2019). In addition to this, *vrs4* mutants show, similar to *hvtb2,* an outgrowth of lateral branches (Koppolu et al. 2013; Zwirek et al. 2019). In this respect, it is interesting to note that *hvtb2* did not display an obvious six-rowed phenotype. In line with this, our RT-PCR analysis showed that *VRS1* expression was not significantly altered in the *hvtb2* mutants.

In conclusion, the protein–protein interaction studies of VRS5 resulted into the identification of HvTB2 as putative interactor of VRS5. Our analysis and two additional recently published independent studies (Shang et al. 2020; Poursarebani et al. 2020) have shown the essential role of HvTB2 in maintaining the characteristic unbranched barley spike. In addition, analysis shows that VRS5 has a diverse interaction capacity, interacting with class II TCP’s, NF-Y TF, but also chromatin remodelers. Further, VRS5 preferably interacts with other class II TCP TF within the TB1 clade. Understanding the molecular network, including protein–protein interactions, of key regulators of plant architecture such as VRS5 provide new routes towards the identification of other key regulators of plant architecture in barley.

## Materials and methods

### Phylogenetic analysis

Sequences of Rice, Maize Arabidopsis and Barley TCP TF were downloaded from the iTAK (Zheng et al. 2016) (http://itak.feilab.net/) and GRASSIUS (Gray et al. 2009) (http://www.grassius.org) databases and manually curated for missing TCPs. In addition to this, for barley we performed a BLAST search against the barley genome using the IPK ViroBLAST (https://webblast.ipk-gatersleben.de/) (Deng et al. 2007; Mascher et al. 2017) using all protein sequences downloaded from the iTAK database. This enabled us to verify if we included all barley TCPs in our analysis. Protein sequences were aligned using MUSCLE (Edgar, 2004) in MEGA version 7.

To identify homologs of HvTB2, we performed a blastp search using the protein sequence as query in the Phytozome database (https://phytozome.jgi.doe.gov/) (Goodstein et al. 2012) against peptide sequences from the following species: *Arabidopsis thaliana*, *Brachypodium distachyon*, *Carica papaya*, *Cucumis sativus*, *Hordeum vulgare*, *Medicago truncatula*, *Oryza sativa*, *Populus trichocarpa*, *Ricinus communis*, *Sorghum bicolor*, *Solanum lycopersicum*, *Triticum aestivum*, *Vitis vinifera*, and *Zea mays*. BLAST results were filtered with an E-value cutoff of 1E − 10. The phylogenetic tree of HvTB2 homologues was rooted using *Selaginella moellendorffii* homolog as an outgroup.

Sequences were aligned using MUSCLE (Edgar 2004) in MEGA version 7 (Tamura et al. 2011).A maximum likelihood phylogenetic tree was constructed using RAxML (Stamatakis 2014), using autoMRE for assessing convergence during bootstrapping. For the phylogenetic tree on all TCPs and the HvTB2 homologues only the convergence test was met after 50 and 100 replicates, respectively. The resulting phylogenetic trees were visualized in EMBL iTOL v4 (Letunic and Bork 2019).

### Haplotype analysis

Haplotype analysis was performed as described previously (Walla et al. 2020), using a set of 39 research/breeding lines, 137 landraces and 91 wild barley accessions published previously (Russell et al. 2016). Further exploration of the natural variation was performed by including the WHEALBI dataset (Whealbi), for which only the SNP matrixes are publicly available (Bustos‐Korts et al. 2019).

### Construction of the yeast two-hybrid libraries and protein–protein interaction studies

Barley seedlings, cv Golden Promise (PI 343079, GSHO 1733, https://www.nordgen.org/), were grown under long day (LD) conditions (16 h, 22 °C day; 8 h, 18 °C night). Samples were taken two hours before the end of the light period to maximize the expression of genes involved in floral organ development and flowering time. The developing seedlings were grown in 96-well trays, and fertilized when necessary. Before sampling the development of the main shoot apex (MSA) was scored according to the quantitative scale by Waddington et al. (1983). This scale is based on the progression of the most advanced floret primordium and carpel of the inflorescence. The reproductive MSA is specified by the appearance of the first floret primordia referred to as the double ridge stage (W1.5–W2.0). Subsequently, the first lemma primordium occurs (W3.0) followed by the stamen primordium stage (W3.5), which is characterized by the differentiation of the first floral organ primordia and the stem elongation. The induction of floral organ primordia continues until the awn primordium stage (W5.0). The last stage sampled for the library was W6.0, at this stage the stylar canal is closing. For each stage (W0–W5), at least 10 MSA in three independent biological replicates were pooled. Two Y2H screening libraries were generated one for the early developmental stages (W0–W1.5) and one for the late developmental stages (W2.0–W6). These stages have been selected as *VRS5* is mainly expressed in the developing shoot apex. All MSA harvested for RNA extraction were frozen immediately in liquid nitrogen and stored at − 80 °C. RNA was isolated as described previously (van Esse et al. 2017). Libraries were constructed using the CloneMiner™II kit, according to manufacturer’s protocol. One exception was the propagation of the libraries in *E. coli*, which was done on large 150 mm in diameter petri dishes instead of liquid medium. The pDEST22 vector was used as prey vector, and thus the destination vector for the libraries. The resulting libraries contained a titer of 8.87 × 10^6^ and 1.73 × 106 CFU. The variation of the genes in these libraries has been tested by colony PCR followed by sequencing of the PCR amplicon.

Primers targeting the TCP transcription factors used in the yeast-two hybrid screen are listed in Supplementary Table S6. The corresponding TCPs were amplified using Q5^®^ High-Fidelity DNA Polymerase (New England Biolabs) from the cDNA screening library and cloned into pDONR201. For VRS5 (also referred to as INTERMEDIUM-C) the *int-c.a* and *int-c.b* alleles (Ramsay et al. 2011) were amplified from cDNA of respectively, cv. Morex (CIho 15773, https://www.nordgen.org/) and cv Bowman (PI 483237, https://www.nordgen.org/). Subsequently, the TCP TF were cloned into the bait (pDEST32) and prey (pDEST22) vectors. To prevent auto activation in the bait constructs (pDEST32) the N-terminal part of the full length TB1 protein was removed (VRS5^NtDEL83^). The TCP domain was kept intact for all constructs used. Autoactivation was tested on selective medium containing -L + 3AT and -LA. Only the -LA marker showed no autoactivation (Supplementary Fig. 4A), and therefore the screen was performed using -LWA medium. As negative control, HORVU.MOREX.r2.3HG0240550 was included, which is annotated as a transcriptional regulator without known domains. As positive control, all plates were grown on media containing -LW in parallel to the selective -LWA plates. For the heterologous screen against the Arabidopsis TF library (Pruneda-Paz et al. 2014), VRS5^NtDEL83^ was used as bait and the library as prey. Subsequently, the screen was performed on -LWA medium using medium containing -LW as positive control, as described above.

### CRISPR-Cas9 mutagenesis

For CRISPR-Cas9 mutagenesis OsU3 promoter, which used a “A” as start site, was used, which was linked in the Golden Gate vector system (Chiasson et al. 2019). In total three guides were used. guide 1: GCAGCTTCTCCATGGCGCCT; guide 2: GCTCCTCCTCTGGCGGACAT; guide 3: ACTGGCGCAGTGCAGGCCGC. Plants were transformed as described previously (Hinchliffe and Harwood 2019). The resulting primary transformants were selected for presence of Cas9. In the second generation, two lines were selected based on mutational events. Transformants were genotyped using the Phire Plant Direct PCR Kit (Thermo Fisher Scientific), amplified fragments were directly sequenced. Primers for genotyping the generated mutants are added in Supplementary Table S6.

### Plant growth and phenotyping

For plant phenotyping cv Golden Promise (GP) and *hvtb2* mutants were grown on soil at 22 °C during the day (light, 16 h) and 16 °C during the night (in darkness, 8 h) in 1 L pots supplied with fertilizer and water when needed. Tiller number was recorded at full maturity. Thousand grain weight (TGW), grain number per spike and size were recorded after drying of the spike/grains. Statistical analyses were performed using the statistical software R (http://www.r-project.org/) Differences between wild-type and mutant genotypes were determined using a student’s *t* test or a one-way ANOVA combined with a Tukey HSD for multiple comparison.

### RNA in situ hybridization and EM microscopy

Plants were grown on soil at 22 °C during the day (light, 16 h) and 16 °C during the night (in darkness, 8 h) in small 40-well trays. Probes for HvTB2 mRNA were prepared from the whole coding sequence (start to stop codon). Cloning and RNA probe synthesis was performed as described previously (Kirschner et al. 2017) and used as full-length RNA probes or with a subsequent hydrolysation to 150 bp. RNA in situ hybridizations on shoot apical meristems of the double ridge stage (and the awn primordium stage were performed as described before (Kirschner et al. 2017).

For scanning electron microscopy, dissected main inflorescences were mounted on a copper specimen holder with freeze hardening glue and frozen in liquid nitrogen. Images were obtained using a FEI Magellan 400 microscope, which is equipped with a Leica cold stage for cryo-microscopy. Low-temperature SEM was performed on the frozen shoot apical meristems. Images of *hvtb2-1* and *hvtb2-2* were processed using Adobe Photoshop to color code the outgrowing side shoots. Staging of the apex over development was done using a standard binocular microscope.

### Expression analysis

Expression analysis of genes interacting with VRS5 was done by re-analyzing the RNA-Sequencing libraries of cvBowman obtained from GSE102191 (van Esse et al. 2017) and GSE149110 (Walla et al. 2020). RNA-Seq data analysis was performed as described previously (Walla et al. 2020). In addition, we used recently published expression data to evaluate the expression of VRS5, HvTB2 and other TCPs (presented in Fig. S1) using publicly available data sets (Thiel et al. 2021).

For RT-PCR and monitoring the shoot apex development, plants were grown in 96-well trays, under controlled greenhouse conditions similar to the conditions used for the RNA in situ hybridization and EM microscopy. Developing inflorescences were collected at the lemma and stamen and awn primordium stages. The last leaf surrounding the apex was not removed, as such the material was leaf enriched. RNA-isolation for RT-PCR analysis was done using the PureYield™ RNA kit (Promega). Subsequently, cDNA was generated using the iScript cDNA synthesis kit (Biorad) using 1 µg of RNA for each cDNA reaction. The cDNA was diluted 5 times, and RT-PCR was performed using iQ™ SYBR^®^ Green Supermix (Biorad) following manufactures protocol. The SYBR signal was detected using a Bio Rad MyiQ. The resulting CT values were used to calculate ${2}^{{\Delta \Delta C_{{\text{T}}} }}{2}^{{\Delta \Delta C_{{\text{T}}} }}$ values, using HvADP as a control gene. Primers for RT-PCR analysis are included in Supplementary Table S6, and were as previously reported for HvADP (Walling et al. 2018); VRS1 (van Esse et al. 2017); VRS3, VRS4, VRS5 and HvIDS (Zwirek et al. 2019); VRS2 (Youssef et al. 2017) and SPL8.1 (Tripathi et al. 2018). All RT-PCR experiments were done in at least three biological replicates. Statistical differences were determined using a two-tailed unpaired Student’s *t* test.

### Plant materials

Seeds from *intermedium-h* and *compositum* were ordered at the Nordic gene bank (https://www.nordgen.org/). The following mutants were used: *int-h.42* (NGB20658); *int-h.43* (NGB115461); *int-h.44* (NGB115462); *int-h.83* (NGB115501); *com1.a* (NGB22021); *com1.a* NGB116495); *com1.b* (NGB116496); *com1.c* (NGB116498); *int-k.47* (NGB115465); *com3.h* (NGB22025); *com1.d* (NGB116433) *com1.e* (NGB116442); *com1.i* (NGB22022); *com1.1 (*NGB22171); *com 1.j*(NGB22020); *com1.l* (NGB22370) Parental lines Bowman (PI 483237) and Bonus (NGB 14657, PI 189763).

#### Author contribution statement

TSM, WvE, SWvE, and SRdS performed protein–protein interaction studies. FvdW, JB and WvE generated Y2H screening libraries. WvE and TSM selected CRISPR–CAS lines and performed phenotypical analysis of CRISPR lines. WvE and TSM performed phylogenetic and haplotype analysis and genotyping of the *com1* and *int-h* lines. GK performed the in situ hybridization. MM and IHP generated CRISPR–Cas9 mutants. GCA, RGHI, WvE, conceived and designed research and wrote the manuscript with contributions from all co-authors.

## Supplementary Information

Below is the link to the electronic supplementary material.

Supplementary file1 (XLSX 73 kb)

Supplementary file2 **Supplementary Fig. 1** Expression of TCP TF in different developmental stages and tissue types. Overall, TCPs are expressed in various tissue types and developmental stages. Expression data are based on publicly available datasets (Thiel et al. 2021), values are transcript per million (TPM) (TIF 21881 kb)

Supplementary file3 **Supplementary Fig. 2** Maximum likelihood phylogenetic tree of HvTB2-like genes in 19 monocot and eudicot plant species. Tree was built using the protein sequences. The sequence of a TCP homolog obtained from Selaginella moellendorffii (transcript ID 89227) was used for rooting. The barley HvTB2 gene described in this study, HvTB2, is highlighted in red. Functionally characterized genes within the same clade are marked in blue. Arabidopsis class II TCPs in the eudicot branched are marked in green. Bootstrap support (%) is shown at the nodes. Abbreviated species names are given before gene identifiers. Aet: Aegilops tauschii; Ath: Arabidopsis thaliana; Bd: Brachypodium distachyon; Cp: Carica papaya; Cs: Cucumis sativus; Hv: Hordeum vulgare; Mt:Medicago truncatula; Os: Oryza sativa; Pt: Populus trichocarpa; Pv: Phaseolus vulgaris; Rc: Ricinus communis; Sb: Sorghum bicolor; Sc: Secale cereale; Si: Setaria italica; Sl: Solanum lycopersicum; Ta: Triticum aestivum; Vv: Vitis vinifera; Zm: Zea mays. Scale bar = 0.1 substitutions per site (TIF 77039 kb)

Supplementary file4 **Supplementary Fig. 3** Barley apex and crown tissue used isolated to generate yeast two-hybrid libraries. Main shoot apex (MSA) and crown tissue of developing barley seedlings was excised at different developmental stages. Library 1 was made from crown tissue including the vegetative apical meristem. Library 2 was made from meristem tissue obtained during various stages of floral organ development, starting at the floral transition which is marked by the double ridge. The last samples for library 2 were taken after the induction of floral organ primordia was completed. Random PCR amplification of the inserts present in several independent colonies. indicated that the libraries include cDNA fragments between 500 and 2000 bp. Sequencing of 10 colonies verified that there was a good variation in the identified proteins (TIF 1234 kb)

Supplementary file5 **Supplementary Fig. 4** Detailed overview of yeast-two-hybrid protein–protein interactions. (a) Table showing the results of the autoactivation test. For each construct at least three colonies were scored. (b) Number of replicates performed in the protein–protein interaction studies (top panel), compared to the number of interactions observed (middle and bottom panel). Each interaction was scored in at least 6 independent replicates. Differences between replicates are visualized by dividing the interactions scored by the number of replicates with: a score of 0 (yellow) no interaction; and a score of 1 (dark green) always an interaction; values in-between 0 and 1 indicate the constancy of the results between replicates. (TIF 18159 kb)

Supplementary file6 **Supplementary Fig. 5** Target region for CRISPR–CAS mutagenesis of HvTB2. Black bars mark the three guides, red triangles the NGG PAM recognition site. The orange block shows the TCP domain. (TIF 33364 kb)

Supplementary file7 **Supplementary Fig. 6** Phenotypical analysis of the hbtb2 mutant. (a) Original images used to generate the subpanel 3B. Three representative seeds with awn were selected to visualize the difference in awn architecture for seeds on the basal part of hvtb2 mutants when compared to the wildtype cv. Golden Promise (GP). (b) Phenotype of hvtb2 compared to GP, seeds are removed for better visualization of the branches. C) Counting of the fused seed phenotype using the hvtb2-1 mutant. Seed obtained from spikes of three different plants (rep 1,2,3) were evaluated and total number of counted seeds were compared to the number of fused seeds. D–F). Seed parameters TGW (n = 20); grain width (n = 30; and grain length (n = 30). In the hvtb2 mutants seeds from the main spike and the branch were measured separately. Statistical differences are based on a one-way ANOVA, combined with a combined with a Tukey HSD for multiple comparison. Letters indicate differences when compared to GP using a P ≤ 0.05 (TIF 41528 kb)

Supplementary file8 **Supplementary Fig. 7** Genotyping int-h and com1. (a) Table showing the PCR amplification results of int-h and com1 lines. Green marks regions where a PCR amplicon was obtained, gray indicates no amplicon. Top panel shows the regions that were targeted in the PCR analysis: promoter region of HvTB2 (dark green), coding sequence (blue) and downstream region (orange). Up to a region of 2,500 base pairs upstream of the start and 7050 bp downstream of the start no PCR amplicon was obtained in int-h.42, int-h.43 and int-h.44 as well as com1.a and com1.b. (b and c) PCR agarose gels used to construct panel (a). (b) a larger panel of 16 compositum lines and two controls cv Bowman and cv Bonus. For int-k.47, com1.d, com1.e. com1.i. com1.1, and com1.l no mutation was identified in the coding sequence while for int-h42, int-h.43 and int-h.44, int-h.83, com1.a and com1.b no amplicon was obtained. (c) genotyping of int-h42, int-h.43 and int-h.44, int-h.83, com1.a, com1.b and com1,j using a broader range of primer sets to amplify the target region. Primers used in the assay are included in Supplementary Table S6 (TIF 80426 kb)

Supplementary file9 **Supplementary Fig. 8** Genotyping int-h and com1. (a) int-h.83 and com1.c contained a non-synonymous polymorphism within the conserved TCP domain (black box) which was not present in the wild type control nor identified as common haplotype. (b) PROVEAN score, which predicts whether an amino acid substitution or indel has an impact on the biological function of a protein indicates that there is no effect of the observed haplotypes HAP2, 7 and 8 while the SNPs in int-h83 and com1.c are predicted to have deleterious effects (TIF 37955 kb)

Supplementary file10 **Supplementary Fig. 9**. Shoot apical meristem development of hvtb2-1 compared to cv. Golden Promise. (a) development of the shoot apex of wildtype and hvtb2-1 mutant. At double ridge stage no differences were observed while at awn primordium stage a clear outgrowth of the lateral branch is observed. (b) Shoot apical meristem development of cv Golden Promise (GP) versus hvtb2-1, monitored using the Waddington scale (W). (c) leaf number of hvtb2-1 compared to the wildtype GP. For both (B) and (C) n ≥ 6 plants. No significant differences were observed (TIF 24956 kb)

Supplementary file11 **Supplementary Fig. 10** In-situ hybridization in cv Bowman targeting HvTB2. The RNA in situ hybridization was performed at the double ridge stage, (a) and the awn primordium stage (b). The first two images in panel B show the original compiled images used for Fig. 4B, whereas the third image shows the same tissue but a different sectioning depth (TIF 65314 kb)

Supplementary file12 **Supplementary Fig. 11** Haplotype analysis of VRS5. (a) Haplotype network of VRS5 comprising elite, landrace and wild barley lines. In total sequences of 670 different genotypes were included in the analysis. The two major haplotypes HAP1 and HAP2 were observed for VRS5. (b) Representation of the two main VRS5 haplotypes corresponding to the two-rowed and six-rowed cultivars. These correspond to the previously reported int-c.a and int-c.b alleles (Ramsay et al. 2011) (TIF 91666 kb)

## Abbreviations

TCP: TEOSINTE BRANCHED 1, CYCLOIDEA, PCF1
TF: Transcription factor
TB1: TEOSINTE BRANCHED 1
CIN: CINCINNATA
CYC: CYCLOIDEA
SAM: Shoot apical meristem
DR: Double ridge stage
LP/SP: Lemma and stamen primordia stages
AP: Awn primordia stage
Hv: Hordeum vulgare
cv: Cultivar
GP: Golden promise
*int-h*: *Intermedium-h*
*com*: *compositum*
BDI1: BRANCHED AND INDETERMINATE SPIKELET 1
SNP: Single-nucleotide polymorphism
SPL: SQUAMOSA PROMOTER-BINDING-LIKE8
REP1: RETARDED PALEA 1
RA2: RAMOSA
WAB1: Wavy auricle blade 1
BAD1: BRANCHED ANGLE DEFECTIVE 1
msd1: Mutliseeded1
VRS: Six-rowed spike
NF-Y: Nuclear transcription factor Y
SWI/SNF: SWItch/sucrose non-fermentable
