# Supplementary figures and images for: The TCP transcription factor HvTB2 heterodimerizes with VRS5 and controls spike architecture in barley
Source: Plant Reprod. 2022 Mar 7;35(3):205–20. doi: 10.1007/s00497-022-00441-8 (PMC9352630; doi:10.1007/s00497-022-00441-8)

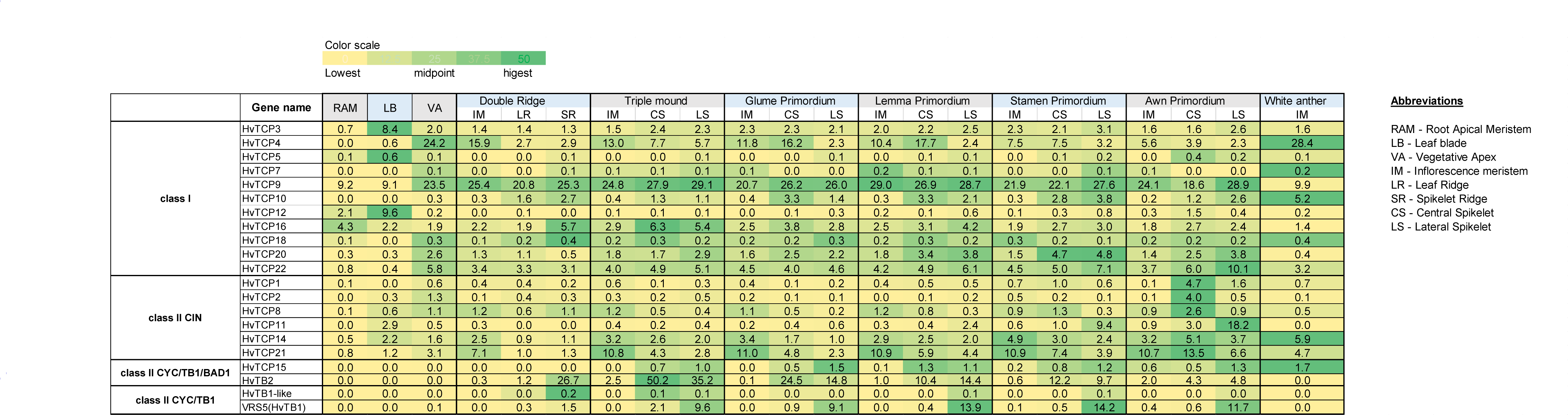

Supplement: Supplementary file 2 — Supplementary file2 Supplementary Fig. 1 Expression of TCP TF in different developmental stages and tissue types. Overall, TCPs are expressed in various tissue types and developmental stages. Expression data are based on publicly available datasets (Thiel et al. 2021), values are transcript per million (TPM) (TIF 21881 kb) [file 497_2022_441_MOESM2_ESM.tif]

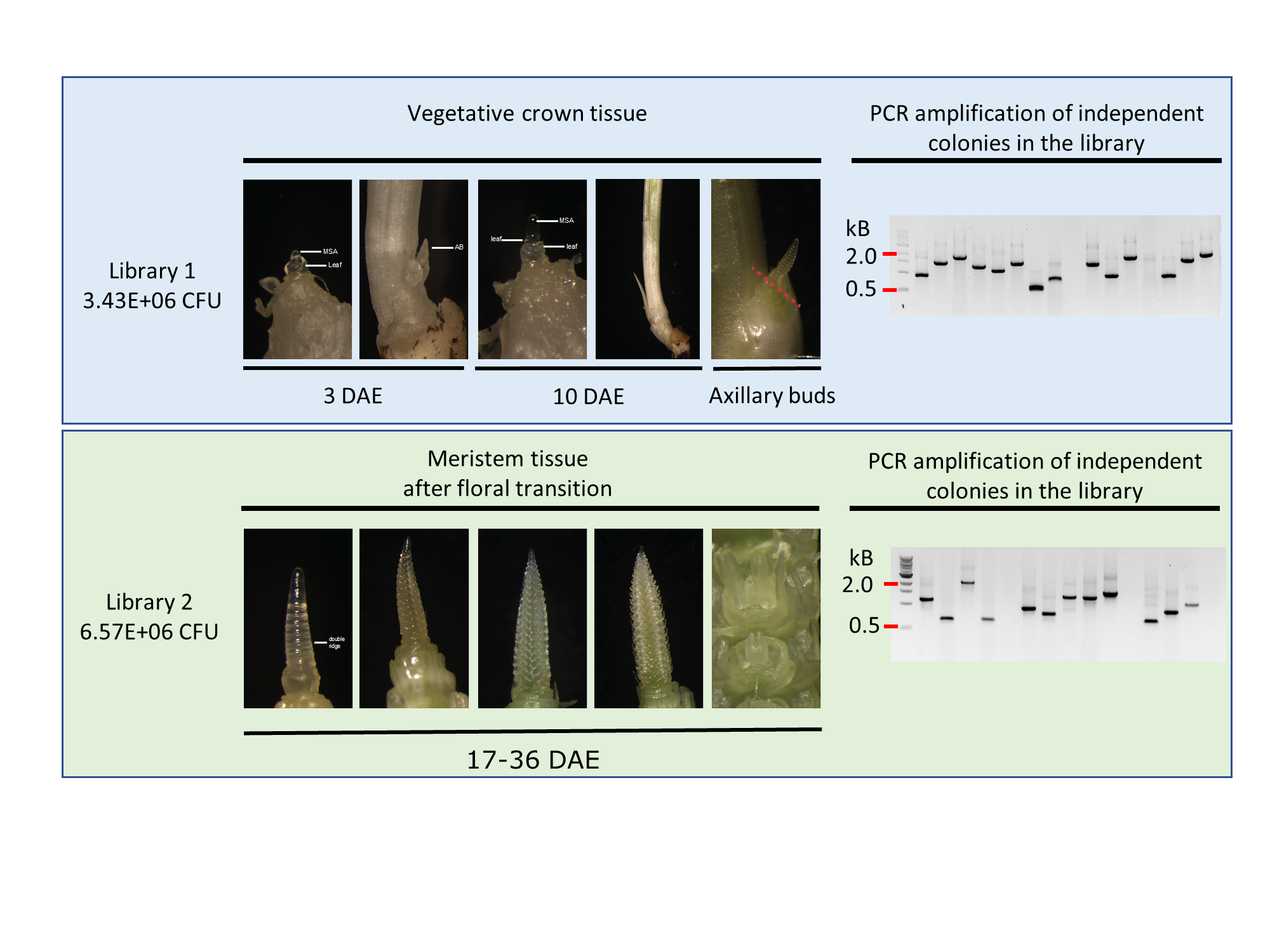

Supplement: Supplementary file 4 — Supplementary file4 Supplementary Fig. 3 Barley apex and crown tissue used isolated to generate yeast two-hybrid libraries. Main shoot apex (MSA) and crown tissue of developing barley seedlings was excised at different developmental stages. Library 1 was made from crown tissue including the vegetative apical meristem. Library 2 was made from meristem tissue obtained during various stages of floral organ development, starting at the floral transition which is marked by the double ridge. The last samples for library 2 were taken after the induction of floral organ primordia was completed. Random PCR amplification of the inserts present in several independent colonies. indicated that the libraries include cDNA fragments between 500 and 2000 bp. Sequencing of 10 colonies verified that there was a good variation in the identified proteins (TIF 1234 kb) [file 497_2022_441_MOESM4_ESM.tif]

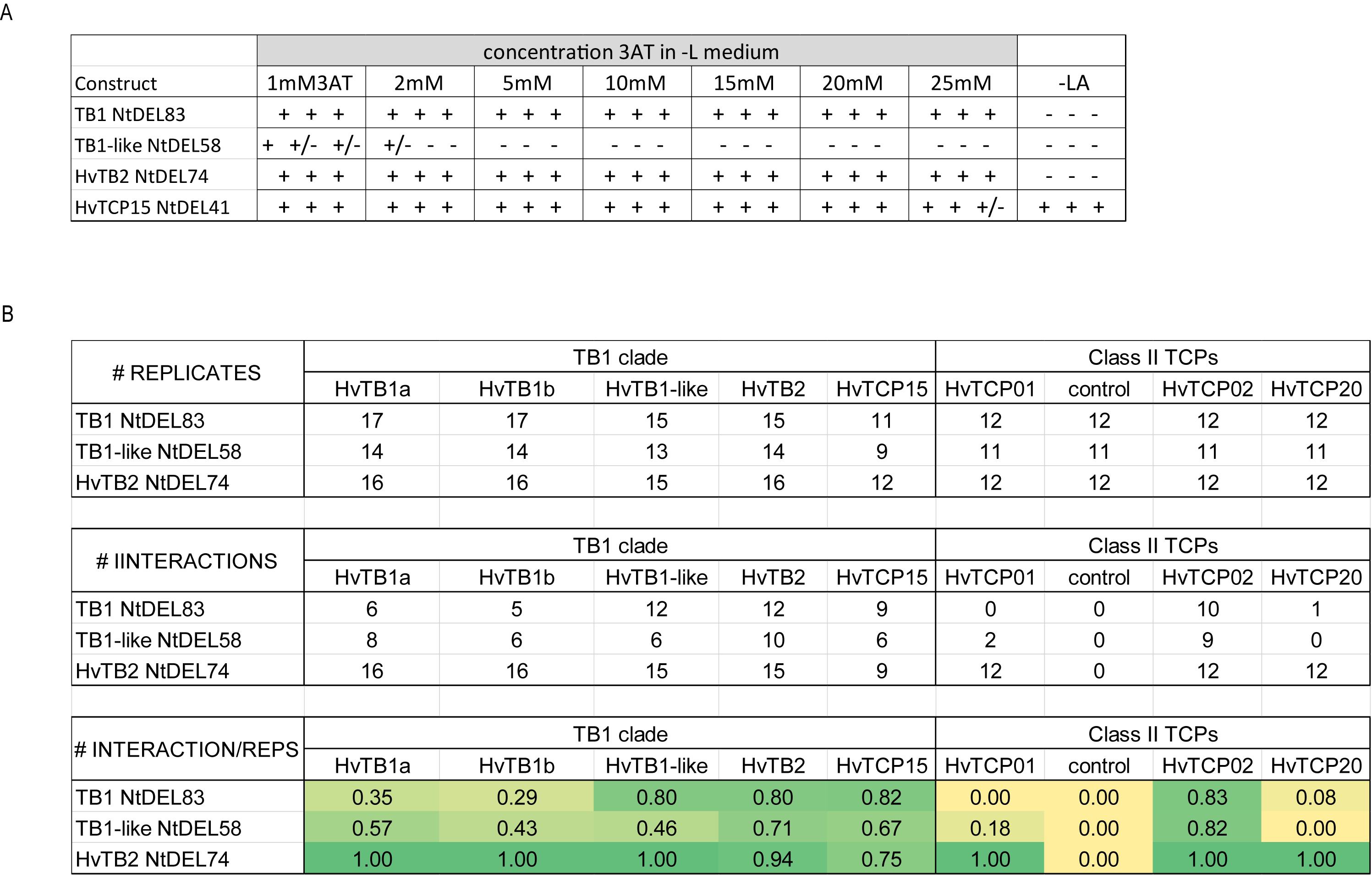

Supplement: Supplementary file 5 — Supplementary file5 Supplementary Fig. 4 Detailed overview of yeast-two-hybrid protein–protein interactions. (a) Table showing the results of the autoactivation test. For each construct at least three colonies were scored. (b) Number of replicates performed in the protein–protein interaction studies (top panel), compared to the number of interactions observed (middle and bottom panel). Each interaction was scored in at least 6 independent replicates. Differences between replicates are visualized by dividing the interactions scored by the number of replicates with: a score of 0 (yellow) no interaction; and a score of 1 (dark green) always an interaction; values in-between 0 and 1 indicate the constancy of the results between replicates. (TIF 18159 kb) [file 497_2022_441_MOESM5_ESM.tif]

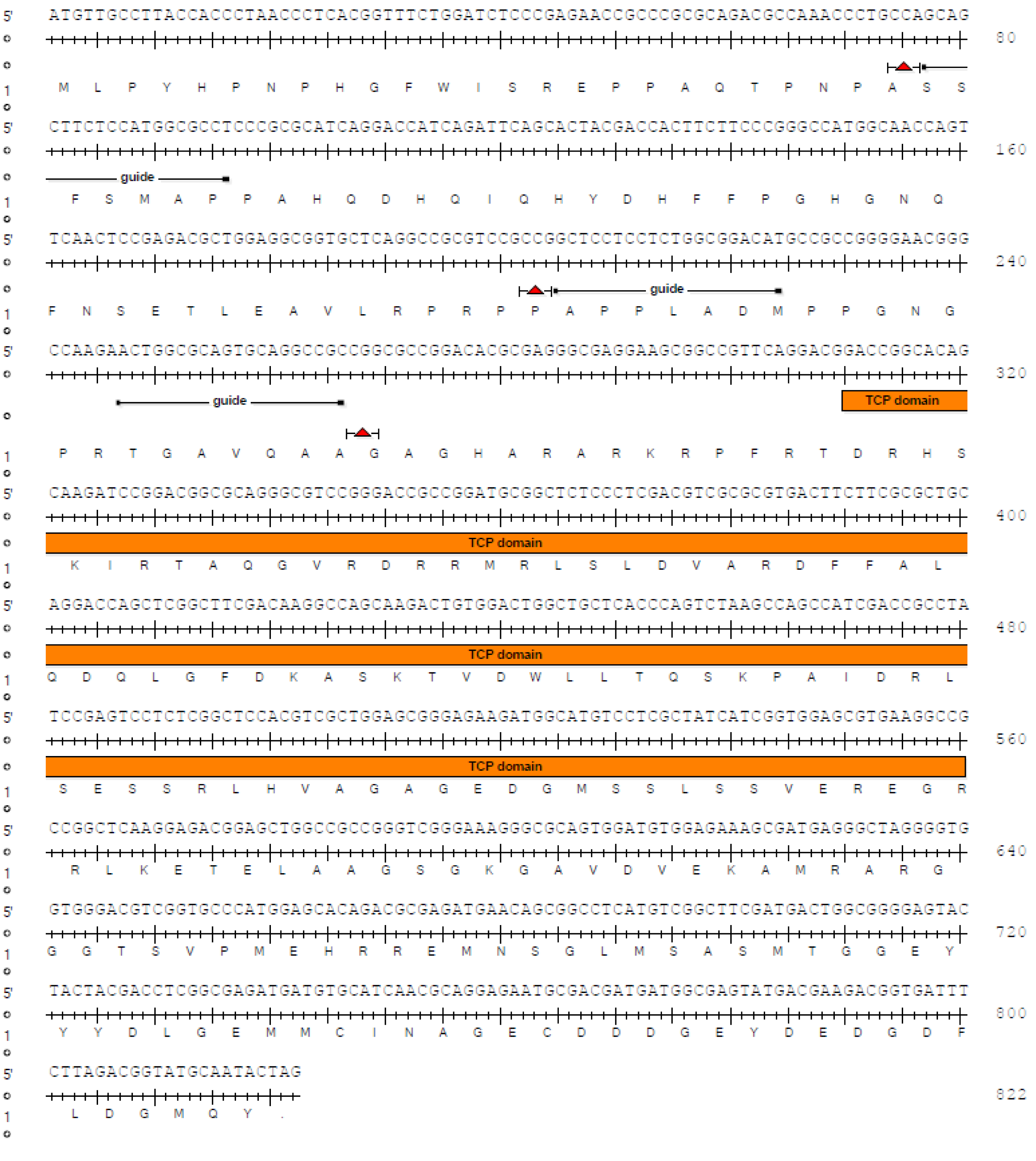

Supplement: Supplementary file 6 — Supplementary file6 Supplementary Fig. 5 Target region for CRISPR–CAS mutagenesis of HvTB2. Black bars mark the three guides, red triangles the NGG PAM recognition site. The orange block shows the TCP domain. (TIF 33364 kb) [file 497_2022_441_MOESM6_ESM.tif]

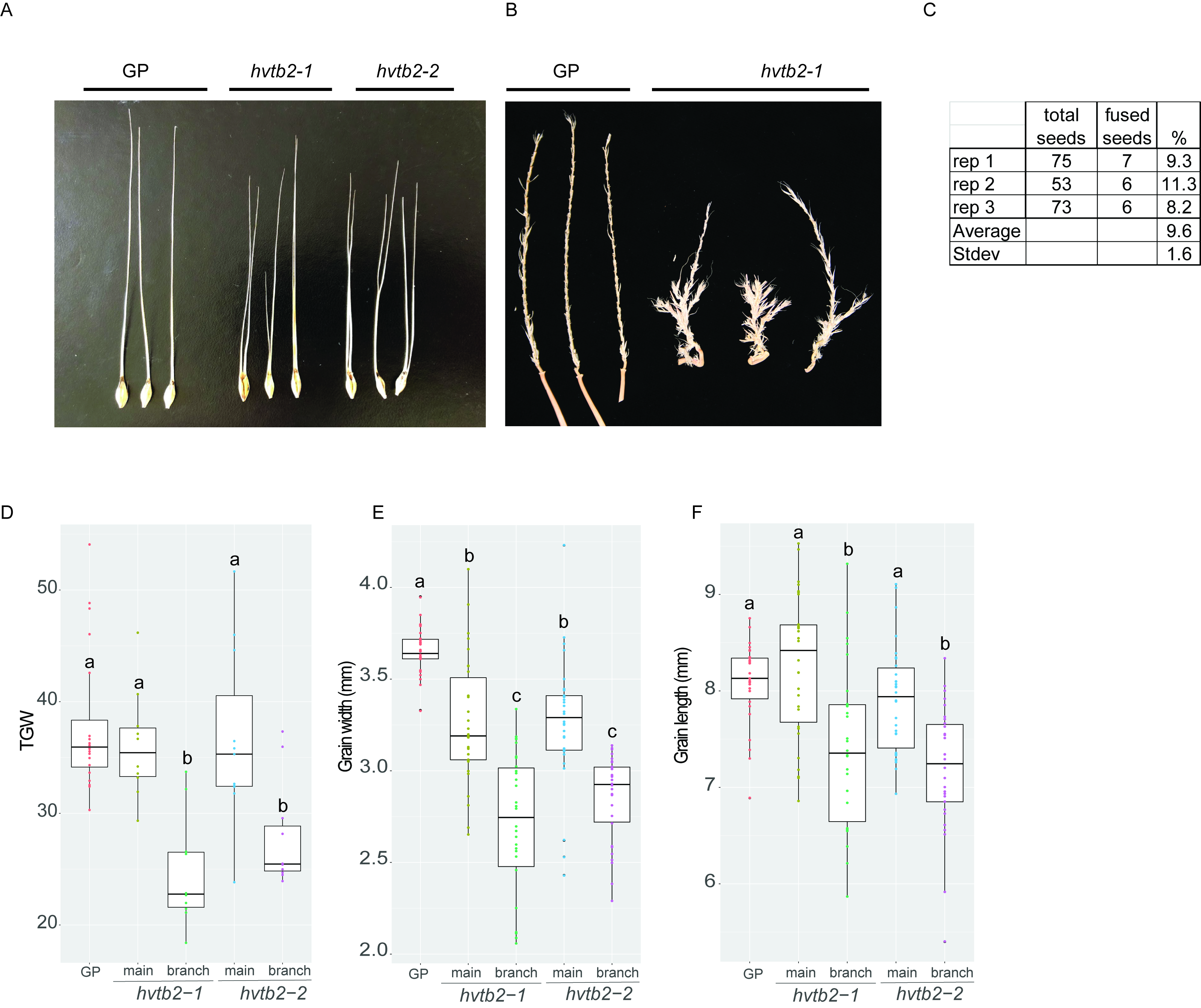

Supplement: Supplementary file 7 — Supplementary file7 Supplementary Fig. 6 Phenotypical analysis of the hbtb2 mutant. (a) Original images used to generate the subpanel 3B. Three representative seeds with awn were selected to visualize the difference in awn architecture for seeds on the basal part of hvtb2 mutants when compared to the wildtype cv. Golden Promise (GP). (b) Phenotype of hvtb2 compared to GP, seeds are removed for better visualization of the branches. C) Counting of the fused seed phenotype using the hvtb2-1 mutant. Seed obtained from spikes of three different plants (rep 1,2,3) were evaluated and total number of counted seeds were compared to the number of fused seeds. D–F). Seed parameters TGW (n = 20); grain width (n = 30; and grain length (n = 30). In the hvtb2 mutants seeds from the main spike and the branch were measured separately. Statistical differences are based on a one-way ANOVA, combined with a combined with a Tukey HSD for multiple comparison. Letters indicate differences when compared to GP using a P ≤ 0.05 (TIF 41528 kb) [file 497_2022_441_MOESM7_ESM.tif]

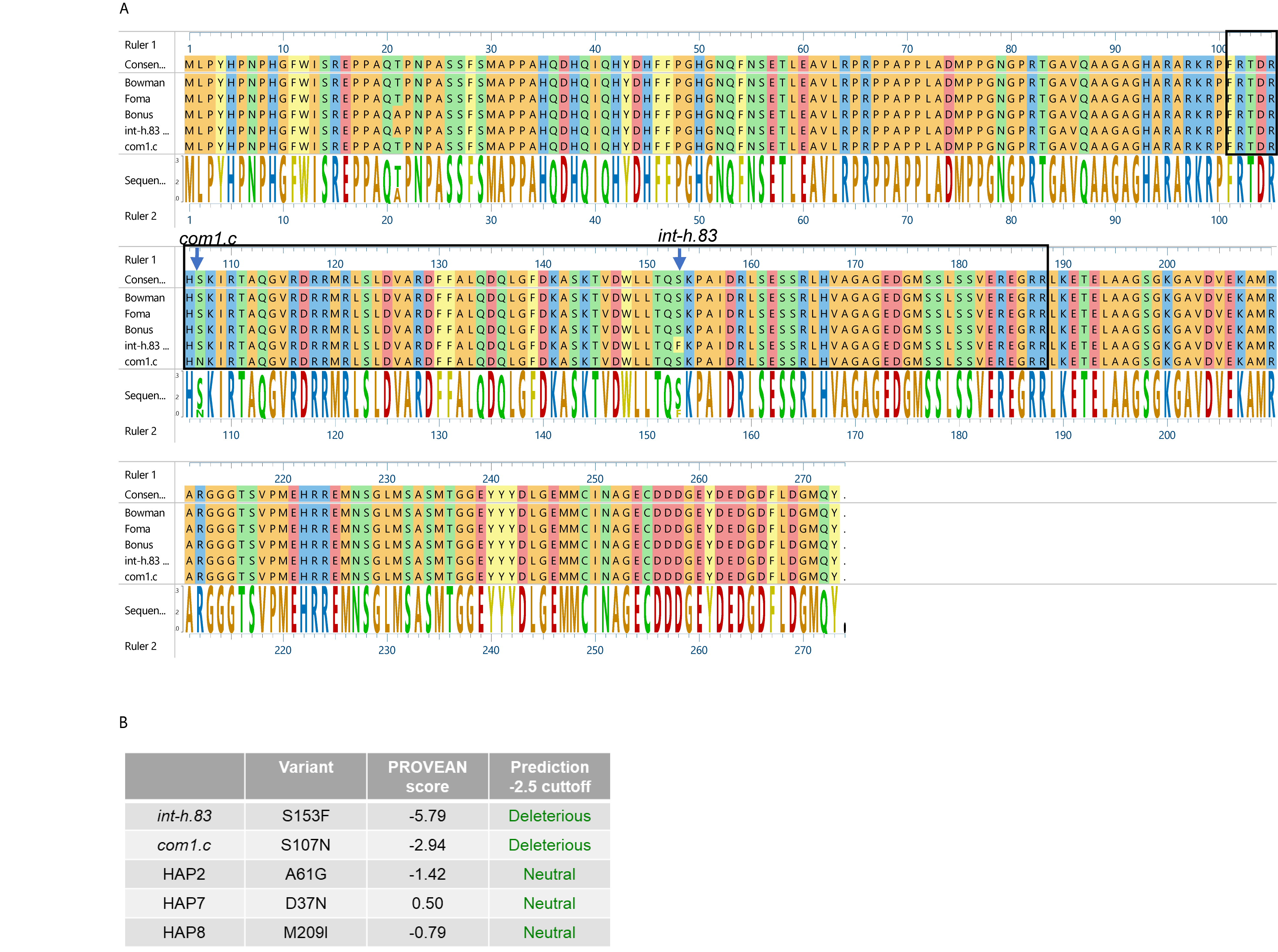

Supplement: Supplementary file 9 — Supplementary file9 Supplementary Fig. 8 Genotyping int-h and com1. (a) int-h.83 and com1.c contained a non-synonymous polymorphism within the conserved TCP domain (black box) which was not present in the wild type control nor identified as common haplotype. (b) PROVEAN score, which predicts whether an amino acid substitution or indel has an impact on the biological function of a protein indicates that there is no effect of the observed haplotypes HAP2, 7 and 8 while the SNPs in int-h83 and com1.c are predicted to have deleterious effects (TIF 37955 kb) [file 497_2022_441_MOESM9_ESM.tif]

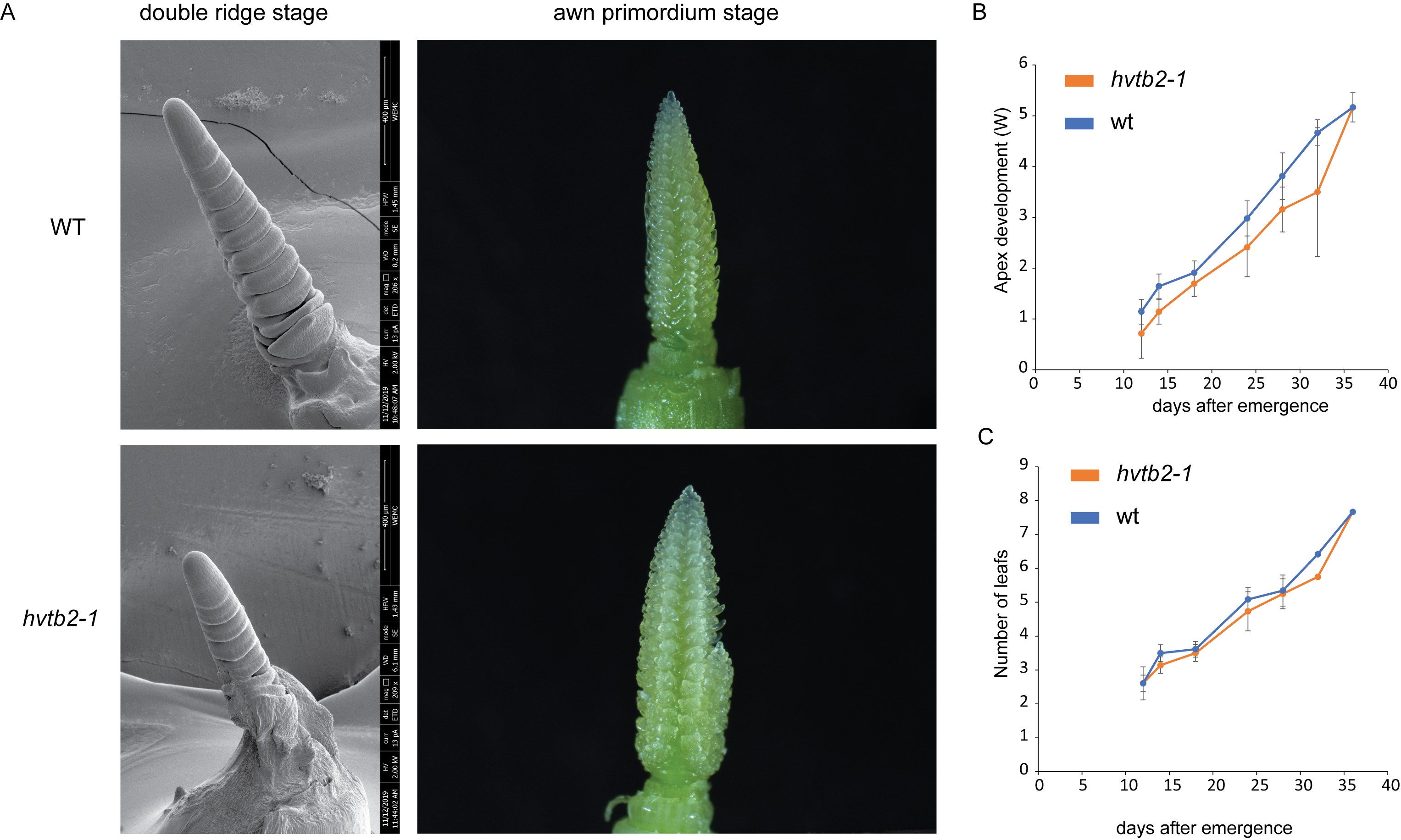

Supplement: Supplementary file 10 — Supplementary file10 Supplementary Fig. 9. Shoot apical meristem development of hvtb2-1 compared to cv. Golden Promise. (a) development of the shoot apex of wildtype and hvtb2-1 mutant. At double ridge stage no differences were observed while at awn primordium stage a clear outgrowth of the lateral branch is observed. (b) Shoot apical meristem development of cv Golden Promise (GP) versus hvtb2-1, monitored using the Waddington scale (W). (c) leaf number of hvtb2-1 compared to the wildtype GP. For both (B) and (C) n ≥ 6 plants. No significant differences were observed (TIF 24956 kb) [file 497_2022_441_MOESM10_ESM.tif]

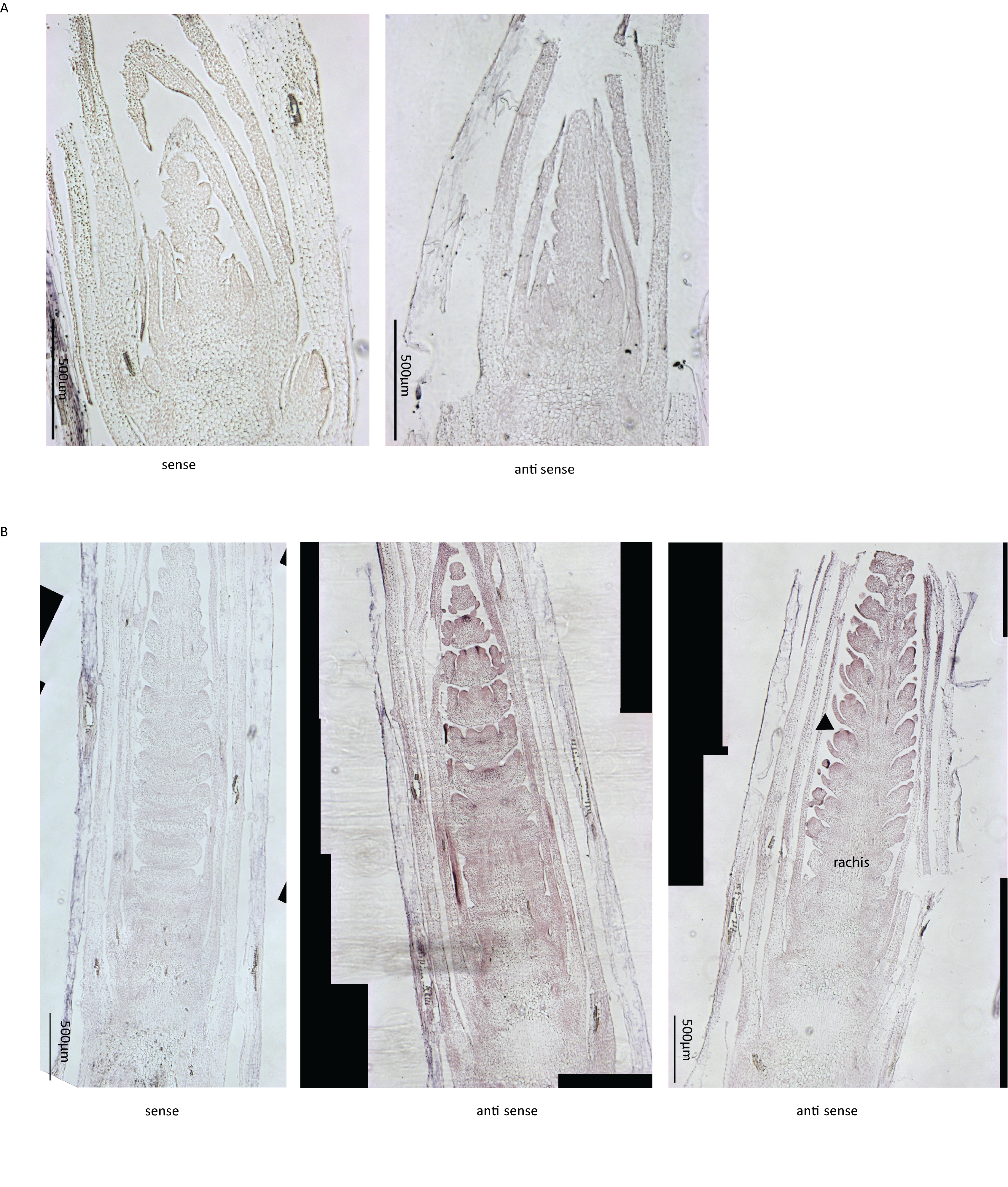

Supplement: Supplementary file 11 — Supplementary file11 Supplementary Fig. 10 In-situ hybridization in cv Bowman targeting HvTB2. The RNA in situ hybridization was performed at the double ridge stage, (a) and the awn primordium stage (b). The first two images in panel B show the original compiled images used for Fig. 4B, whereas the third image shows the same tissue but a different sectioning depth (TIF 65314 kb) [file 497_2022_441_MOESM11_ESM.tif]
